# Microcapsules of Poly(butylene adipate-*co*-terephthalate) (PBAT) Loaded with Aliphatic Isocyanates for Adhesive Applications

**DOI:** 10.1021/acsapm.4c00033

**Published:** 2024-05-02

**Authors:** António Aguiar, Lucas P. Marcelino, António Mariquito, Carla L. Simões, Ricardo Simoes, Isabel Pinho, Ana C. Marques

**Affiliations:** † CERENA, DEQ, Instituto Superior Técnico, Universidade de Lisboa, Avenida Rovisco Pais, 1049-001 Lisboa, Portugal; ‡ Polytechnic Institute of Cavado and Ave (IPCA), 4750-810 Barcelos, Portugal; § Institute for Polymers and Composites (IPC), University of Minho, 4800-058 Guimarães, Portugal; ∥ CIPADE, Av. Primeiro de Maio 121, 3700-227 São João da Madeira, Portugal

**Keywords:** PBAT, microcapsules, isocyanates, HDI, adhesives, environmental assessment

## Abstract

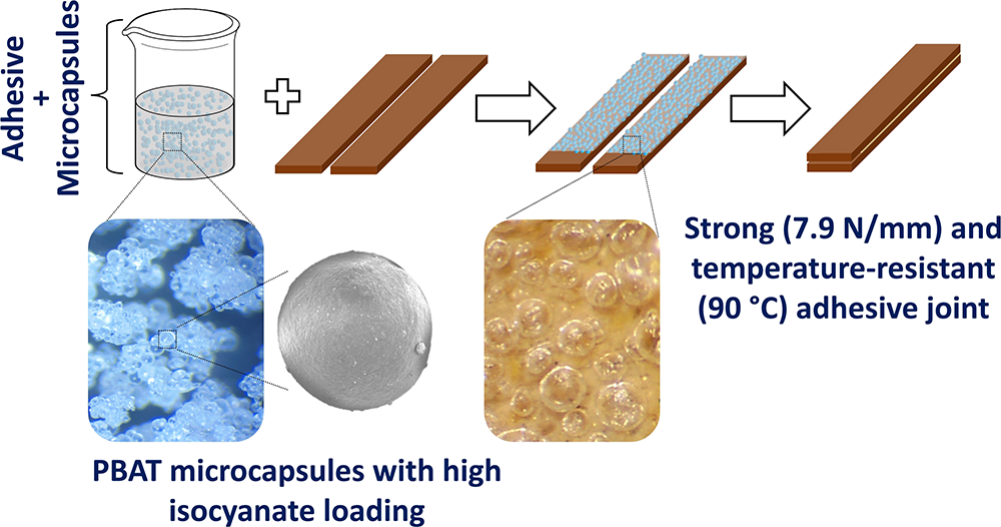

This work introduces the encapsulation of hexamethylene diisocyanate derivatives (HDI, TriHDI, and PHDI) with the biodegradable polymer poly(butylene adipate-*co*-terephthalate) (PBAT) through a solvent evaporation method. These microcapsules (MCs) were then employed in adhesive formulations for footwear. Moreover, MCs containing PHDI were produced in a closed vessel, demonstrating the potential for recovering and reusing organic solvents for the first time. The MCs were achieved with an isocyanate payload reaching up to 68 wt %, displaying a spherical shape, a core–shell structure, and thin walls without holes or cracks. The application of MCs as cross-linking agents for adhesives was evaluated following industry standards. The adhesives’ strength surpassed the minimum requirement by a significant margin. Creep tests demonstrated that the formulation with MCs exhibits superior thermostability. Furthermore, the formulation with MCs-PHDI presented the best results reported to date for this type of system, as no displacement was observed in the bonded substrates. Environmental assessment indicates that adhesives with MCs have higher global warming potential (+16.2%) and energy consumption (+10.8%) than the standard commercial adhesives, but under alternative realistic scenarios, the differences can be insignificant. Therefore, adhesive formulations incorporating MCs promise to be on par with traditional adhesive systems regarding environmental impacts while providing benefits such as improved and safe handling of isocyanates and excellent bonding effectiveness.

## Introduction

Isocyanates represent a highly reactive class of chemical compounds with at least one isocyanate (–N=C=O) group. Methylene diphenyl diisocyanate (MDI), toluene diisocyanate (TDI), hexamethylene diisocyanate (HDI), and isophorone diisocyanate (IPDI) are among the most used isocyanates and are commonly combined with other compounds to form polymeric materials, mostly based on urethane and urea bonds.^1,2^ The versatility of isocyanates makes them suitable for various industries, including manufacturing polyurethane foams, coatings, adhesives, sealants, and elastomers.^3−7^ Due to isocyanates’ structural diversity, and depending on the formulation, processing methods, and additives, the resulting materials can exhibit an extensive range of properties like the strength, durability, flexibility, water resistance, or electrical insulation.^7−10^

Currently, isocyanates hold a crucial position within the adhesive industry, especially in footwear manufacturing. They find utility in crafting footwear soles, creating polyurethane adhesives, and serving as hardener/cross-linker agents.^11^ Isocyanate-based hardeners are commonly mixed with polyurethane or polychloroprene adhesive, two-component adhesive, where the isocyanate must be added to the adhesive immediately before the application.^12^ The cross-linking process reduces the adhesive’s thermoplasticity and improves mechanical stress resistance and resistance to chemicals and solvents.^11^

Despite all of the positive features, the utilization of isocyanates comes with significant health risks for workers involved in the handling of this sort of reagent. Exposure to isocyanates can result in skin irritation and potentially lead to the development of respiratory conditions like asthma.^13,14^ In fact, substances containing more than 0.1 wt % free diisocyanate compounds are subject to restrictions under the REACH regulation unless a safe handling system is implemented.^15^ Microencapsulation of isocyanate species presents a valuable alternative, offering both protection and performance benefits. This solution not only safeguards workers and end-users but also enhances the stability of isocyanates, which is crucial for their storage and transportation.

Microencapsulation is a process that involves enclosing small particles or droplets of a substance within a protective shell, resulting in the formation of microscopic capsules. Its applications span across various fields, each benefiting from its unique advantages. In the food industry, microencapsulation is employed to protect flavors or nutrients, preserving their quality and enhancing their functionality,^16,17^ whereas in the pharmaceutical field, it enhances drug delivery methods.^18^ In agriculture, microencapsulation finds utility in protecting agrochemicals, enabling controlled release mechanisms for enhanced crop management.^19,20^ Microcapsules (MCs) can also indicate damage in materials^21^ or incorporate self-healing and anticorrosion agents.^22,23^ Furthermore, microencapsulation offers the potential to enhance the stability and performance of adhesives by encapsulating active compounds such as curing agents or active resins.^24−26^

The use of diisocyanates in the formation of microcapsule shells through interfacial polymerization has been a common practice.^27^ However, the isocyanate encapsulation is not as usual, and the studies that describe it through interfacial polymerization typically reveal elaborated procedures and nonbiodegradable products.^28^ The emulsion-solvent evaporation technique offers a straightforward, versatile, and efficient approach for encapsulating various materials, including isocyanates, using biodegradable polymers.^29−32^ By adjusting the parameters of the process, such as the polymer concentration, emulsifier, mixing rate, and solvent, it is possible to produce particles with a low size diameter and different morphologies.^33,34^

This paper describes the production of poly(butylene adipate-*co*-terephthalate) (PBAT) MCs loaded with different isocyanate species. PBAT, as a polymeric shell material, offers a range of benefits, including toughness and high elongation at break.^35^ The PBAT’s melting temperature exceeding 100 °C results in PBAT MCs having a substantially higher temperature resistance than other published MCs.^36^ This improvement in isocyanate protection not only enhances product performance but also alleviates concerns related to the transportation and storage of the product, thereby contributing to a more marketable and feasible commercialization perspective. However, there is a scarcity of the literature discussing the use of PBAT for microencapsulation purposes.^29,37^

The MC production process employed in this study was used to encapsulate the hexamethylene diisocyanate monomer (HDI), hexamethylene diisocyanate trimer (TriHDI), and hexamethylene diisocyanate prepolymer (PHDI). The encapsulation of HDI already presents significant advantages due to its substantial vapor pressure (1.4 Pa), and to the best of our knowledge, this study marks the first attempt at encapsulating an HDI polymer derivative by any technique. This work aimed to conduct a comparative assessment of the encapsulation of these aliphatic isocyanates, achieving spherical, well-dispersed, and core–shell MCs. The MCs were then integrated into adhesive formulations, and the resulting adhesive joints were evaluated to determine the feasibility of attaining high strength and thermal resistance levels, while avoiding direct handling with isocyanates. Additionally, for the first time, environmental assessments were conducted for this type of adhesive formulation (adhesive plus MCs) and compared with a commercial adhesive formulation.

## Materials and Methods

### Materials

HDI, TriHDI, and PHDI were supplied by Covestro AG (Leverkusen, Germany) with the commercial names of Desmodur H, Desmodur N3800, and Desmodur DN, respectively. Dichloromethane (DCM), >99.8%, HPLC grade, was obtained from Fisher Chemical (Porto Salvo, Portugal). PBAT (PBAT A400; Mw = 98 000 g/mol) was supplied by Kingfa Sci. & Tech. Co., Ltd. (Guangdong, China). The emulsifier poly(vinyl alcohol) (PVA) (98–99% hydrolyzed, 57,000–67,000 Da) was obtained from Alfa Aesar (Haverhill, MA). The antifoam agent was SARCOFOAM SDQW, sourced from SAPEC Quimica (Setubal, Portugal). All other solvents and products were obtained from Sigma-Aldrich (St. Louis). All of the products were used without further purification. The polyurethane-based adhesive has the commercial name Plastik 6275 and was supplied by CIPADE S.A. (Sao João da Madeira, Portugal). Newcomp—Componentes p/Calçado, Lda (São João da Madeira, Portugal) provided the neolite substrates.

### Microcapsules (MCs) Production

The MCs were produced by emulsion combined with solvent evaporation method, following a previously reported approach with minor adjustments.^29^ Briefly, at room temperature, 3.25 g of PBAT was dissolved in 19 mL of DCM and magnetically stirred until it formed a clear solution. Then, the PBAT solution was added to 6.8 g (31 mmol) of isocyanate species and mixed at 750 rpm for 10 min, forming the organic phase (Oph). The water phase (Wph) was a solution of 3 wt % PVA. Then, the Oph was dispersed in the Wph under mechanical stirring (750 rpm). The emulsion system was left under mechanical stirring for 3 h, and then, the solid MCs were filtered, washed with deionized water several times, and dried in air for 24 h. Then, the MCs were stored in a desiccator at room temperature.

**Figure 1:**
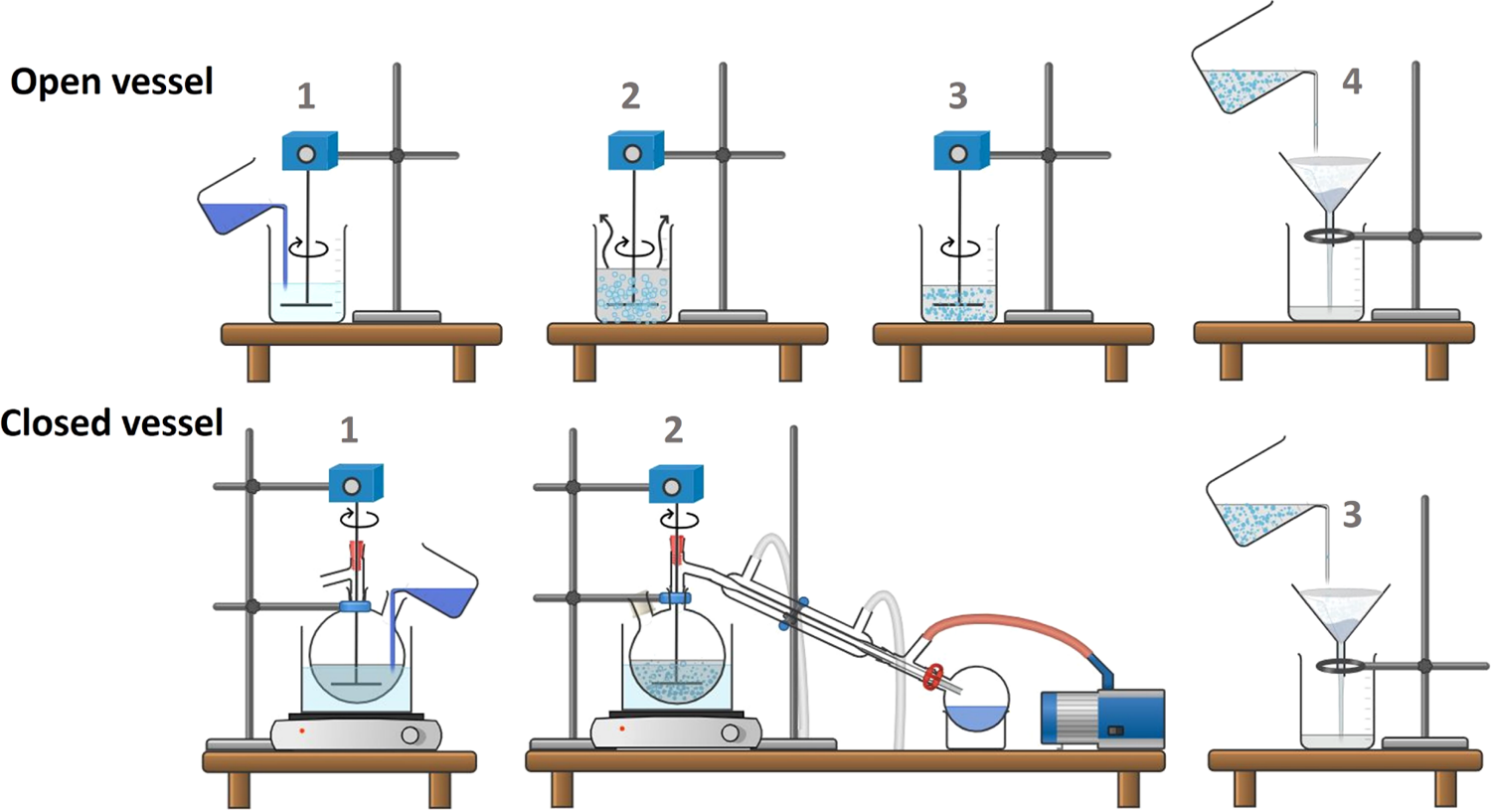
Production of microcapsules by open vessel: (1) addition of the organic phase to the aqueous phase; (2) emulsification and evaporation of DCM; (3) formation of solid MCs; and (4) filtration. Production of microcapsules by closed vessel. (1) addition of the organic phase to the aqueous phase; (2) emulsification and evaporation of DCM at 35 °C and a vacuum of 300 mbar; and (3) filtration.

For assessing solvent recovery, the open-vessel procedure was modified to a closed-vessel setup in which the phase mixing, emulsification, and solvent evaporation stages occurred within a sealed environment, as illustrated in Figure 1. The preceding and subsequent steps were executed exactly as reported for the open vessel. Emulsification and DCM evaporation were conducted at 35 °C and under a vacuum of approximately 300 mbar. An antifoam agent (0.5 wt %) was added to the aqueous phase to manage the foam formation that results from agitation and temperature.

The MCs are identified as MCs-HDI, MCs-TriHDI, and MCs-PHDI, depending on the encapsulation of HDI, TriHDI, and PHDI, respectively. The production yield was calculated by eq 1, where “*m*_total_” is the total weight of PBAT and isocyanate used for MC preparation, and “*m*_MCs_” is the gross weight of recovered MCs
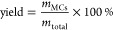
1

### Microcapsules (MCs) Characterization

The MCs’ development, morphology, and surface features were monitored by optical microscopy [Kruss MSZ 5600 optical microscope (Hamburg, Germany)].

MCs’ internal and external morphology was analyzed by scanning electron microscopy (SEM) Phenom ProX G6 benchtop SEM (ThermoScientific, Waltham, MA). The samples were immobilized in a sample holder by using a conductive double-sided adhesive tape and coated with a 15 nm layer of a conductive Au/Pd thin film through sputtering by using a turbomolecular pumped sputter coater from Quorum Technologies, model Q150T ES (Lewes Road, Laughton, U.K.). Microscopy was also used to determine the average diameter of the MCs from data sets of at least 250 measurements. The MCs’ diameters were measured by employing the Fiji software (ImageJ/Fiji 1.53k).^38^ The diameters are represented by the mean (differential size distribution) and *D*_25_, *D*_50_ (median), and *D*_75_ values, which were determined by intercepting 25, 50, and 75% of the cumulative distribution. The mean values are expressed as mean ± standard deviation values.

A Spectrum Two FTIR spectrometer from PerkinElmer (Waltham, MA) equipped with a UATR Two accessory was used. The spectra were obtained at 4 cm^–1^ resolution, and 16 scans of data accumulation were taken.

^1^H NMR spectra were acquired in a Bruker Avance III 400 spectrometer (Karlsruhe, Germany) with CDCl_3_ as the solvent. For isocyanate quantification, an internal standard (4-chloro-3-methylphenol) was employed (signal at 6.50–6.75 ppm), and the signal associated with the –CH_2_NCO group of HDI (3.3 ppm) or –CH_2_N– of TriHDi or PHDI (3.8 ppm) was selected for direct correlation.^40^Equation 2 shows how the isocyanate percentage [Iso (wt %)] was calculated for MCs-HDI and MCs-TriHDI. “*n*_std_” stands for the mol value of the standard, “*k*” is a theoretical ratio between the signal of the used standard and isocyanate when the amount of both is equivalent (*K* = 0.5 for HDI and 0.33 for TriHDI); “*M*_iso_” stands for the molecular weight of the isocyanate; “*r*” is the ratio obtained between the signal of the standard (at 6.50–6.75 ppm) and the signal of the sample (3.3 ppm for HDI or 3.8 ppm for TriHDI); and “mMCs” refers to the total mass of the sample
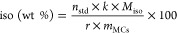
2

As the PHDI is a polymeric compound, the isocyanate was quantified using a calibration curve (Figure S1).

Thermogravimetric analysis [TGA, Hitachi STA 7200 thermal analysis system (Ibaraki, Japan)] was used to study the thermal behavior of the MCs. 5–10 mg of sample was put into an aluminum sample pan and heated from 35 to 600 °C at a rate of 10 °C/min under a nitrogen atmosphere.

The titrations were conducted by adding 0.5 g of MCs to 50 mL of acetone and subjecting the solution to sonication for 20 min. Subsequently, 10 mL of di-*n*-butylamine solution (1 N, toluene) was added and swirled for 30 min. Bromophenol blue indicator solution (3–4 drops) was then introduced. Titrations were carried out using 1 N hydrochloric acid until reaching a yellow color end point. The titration was repeated three times for each sample. Blank titrations were also performed, where all of the reagents mentioned above were used except the MCs. The NCO content is calculated through eq 3, based on ASTM D N2572,^39^ where “*V*_b_” is the volume (mL) of HCl solution required for the titration of 10 mL of di-*n*-butylamine in acetone solution (blank); “*V*_s_” is the volume (mL) of HCl solution required for the titration of the sample; “*C*_HCl_” is the molar concentration of HCl; “*m*” is the weight (g) of the sample, and “42.02” is the molecular weight (g/mol) of the isocyanate group. With the NCO percentage of pure isocyanates (49.7% for HDI; 23% for TriHDI; 21.3% for PHDI) and the “NCO (%)” from the MCs calculated through eq 3, it was possible to achieve the isocyanate wt % MCs by eq 4
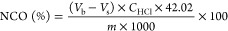
3
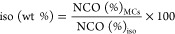
4

### Manufacture and Characterization of a Single Lap Joint

The adhesive formulations comprised Plastik 6275 (component A, commercial polyurethane adhesive) plus encapsulated or nonencapsulated isocyanates (component B, hardener). The quantity of MCs incorporated into the adhesive was adjusted to ensure that the encapsulated isocyanate content constituted 5% of the total weight of the adhesive formulation.

The viscosity measurements of the adhesive formulations were performed in a Modular Compact Rheometer (MCR) Series MCR 92 from Anton Paar (Graz, Austria) with a maximum torque of 125 m·Nm and a minimum torque rotation/oscillation of 1 N·m. Flow tests were performed with a parallel plate system composed of a measuring plate PP25 with a diameter of 25 mm, and a disposable dish EMS/CTD 600 with 54 mm diameter was also used. A gap of 1.00 mm between the measuring plate and the disposable dish was maintained. A shear rate ranging from 1 to 1000 s/1 was applied. From the 21 points taken (at 25 °C), the software interpolated the viscosity value for 50 s^–1^ after accomplishing the flow curves.

Two substrates (13 × 3 cm) of Neolite, synthetic rubber typically used to replace leather, were glued together in an area around 10 × 3 cm. Prior to adhesive application, the substrates were subjected to chemical treatment with 2190 Halinov (CIPADE S.A.) and allowed to dry for 1 h at room temperature. The adhesive formulation was applied on both substrates with a brush and allowed to dry for 15 min at room temperature. The adhesive films were activated by IR radiation at about 70 °C for 6 s, and then the substrates were bonded and subjected to a pressure of 4 bar for 10 s. Then, the adhesive joints were stored for 7 days under standard conditions (23 °C, 50% RH) to ensure the complete cure of the adhesive.

The peel tests were performed following the standard EN 1392 at an angle of 180°. A universal testing machine, Instron 5566 was used at a crosshead speed of 100 mm/min. Three adhesive joint specimens for each test were considered.

The creep strength was evaluated by applying a constant weight (300 g) for 2 h at different temperatures (60–90 °C), to one end of the specimen, while the other end was secured to allow the specimen to be suspended. The displacement was measured every 2 h, followed by a temperature increase of 10 °C. This test is an adaptation of the standard EN 1392:1998. Three adhesive joint specimens were considered for each test.

### Environmental Assessment

The potential environmental impact of a commercial adhesive formulation (CA), composed of Plastik 6275 and nonencapsulated isocyanate (TDI derivative, Cipadur 2230T), and the formulation with Plastik 6275 and MCs-PHDI (MCA) were evaluated using a life cycle assessment (LCA) methodology, which includes all stages of a product’s life.^41−43^ The methodology was performed following the standards from the ISO 14040 series.^44^ This work analyses the global warming potential (GWP), according to the models developed by the Intergovernmental Panel on Climate Change (IPCC) and the energy consumption (nonrenewable fossil) (EC), according to the model developed in the cumulative energy demand (CED) method.^45^ It has been reported that CED correlates well with most environmental indicators,^46,47^ thereby providing an estimate of the environmental impact. A detailed process model was developed based on industrial data, in the case of the polyurethane adhesive, and laboratory data, in the case of the developed formulation, to investigate the environmental impacts associated with these systems and identify the important drivers behind these environmental impacts. The reference unit defined in this study was 1 kg of adhesive produced. A cradle-to-customers’ gate approach was defined. The system boundary includes the raw material extraction and material production, material transportation to the adhesive plant, adhesive production (including microcapsule production in the case of the PUMC adhesive), adhesive packaging production and transport to the adhesive plant, and final adhesive transport to the customer. The life cycle stages of using the adhesive in the footwear industry, shoe, and shoe End of Life (EoL) were not considered in the LCA analysis because it is assumed that these processes are similar in both systems (CA and MCA). All of the systems have been modeled using the commercial Ecoinvent v3.4 database^48^ and, whenever possible, using field data from the companies involved in the study. Some data did not exist in the Ecoinvent database and their inventory was collected from other sources, namely, the European Life Cycle Database (ELCD)^49^ and Industry Data 2.0.^50^ The inventory (Tables S1 and S2) and impact assessment were performed in the SimaPro 8.5 software.^50^

## Results and Discussion

### Production of MCs and Morphology Assessment

During the emulsion-solvent evaporation method, the progress of the emulsion and toughness of the MCs’ shell were continuously monitored using optical microscopy. After 4 h, the mixing was stopped, and the MCs were filtered and washed with water, as they exhibited sufficient resistance to retain their shape during filtration. All syntheses yielded MCs with relatively high yields, specifically 75.7 ± 1.2, 72.7 ± 1.7, and 76.0 ± 2.2% for MCs-HDI, MCs-TriHDI, and MCs-PHDI, respectively. Most of the losses (15–20%) are attributed to the accumulation of some polymer and isocyanate on the reactor walls, mixing impeller, filter, and Buchner funnel.

To ensure the feasibility of the solvent evaporation method in an industrial production setting, it is essential to minimize the release of DCM into the atmosphere. This procedure is crucial for workers’ safety, environmental protection, and economic viability. Considering this, we conducted a single test using the solvent evaporation method for MCs-PHDI production within a closed system, simulating a simple distillation setup and thereby recovering a portion of the DCM. In addition to the agitation parameters and ratios established for open-vessel production, the temperature was set at 35 °C and complemented with a vacuum of 300 mbar. In this first trial, the MCs were solid within 90 min, and around 65% of the DCM was recovered and ready to use in another cycle. The production yield was 70%, and the MCs-PHDI produced through the closed system exhibited aspects similar to those obtained through the open-vessel setup. It has been reported that operational conditions like elevated temperature or reduced pressure can impact efficiency;^51^ however, our experiment revealed a similar level of encapsulation. The purpose of this test was solely to establish the feasibility of solvent recovery and microcapsule formation. Optimizations of this process, particularly concerning parameters such as the production time, temperature, vacuum, and mixing, can potentially enhance production efficiency and facilitate solvent recovery.

**Figure 2:**
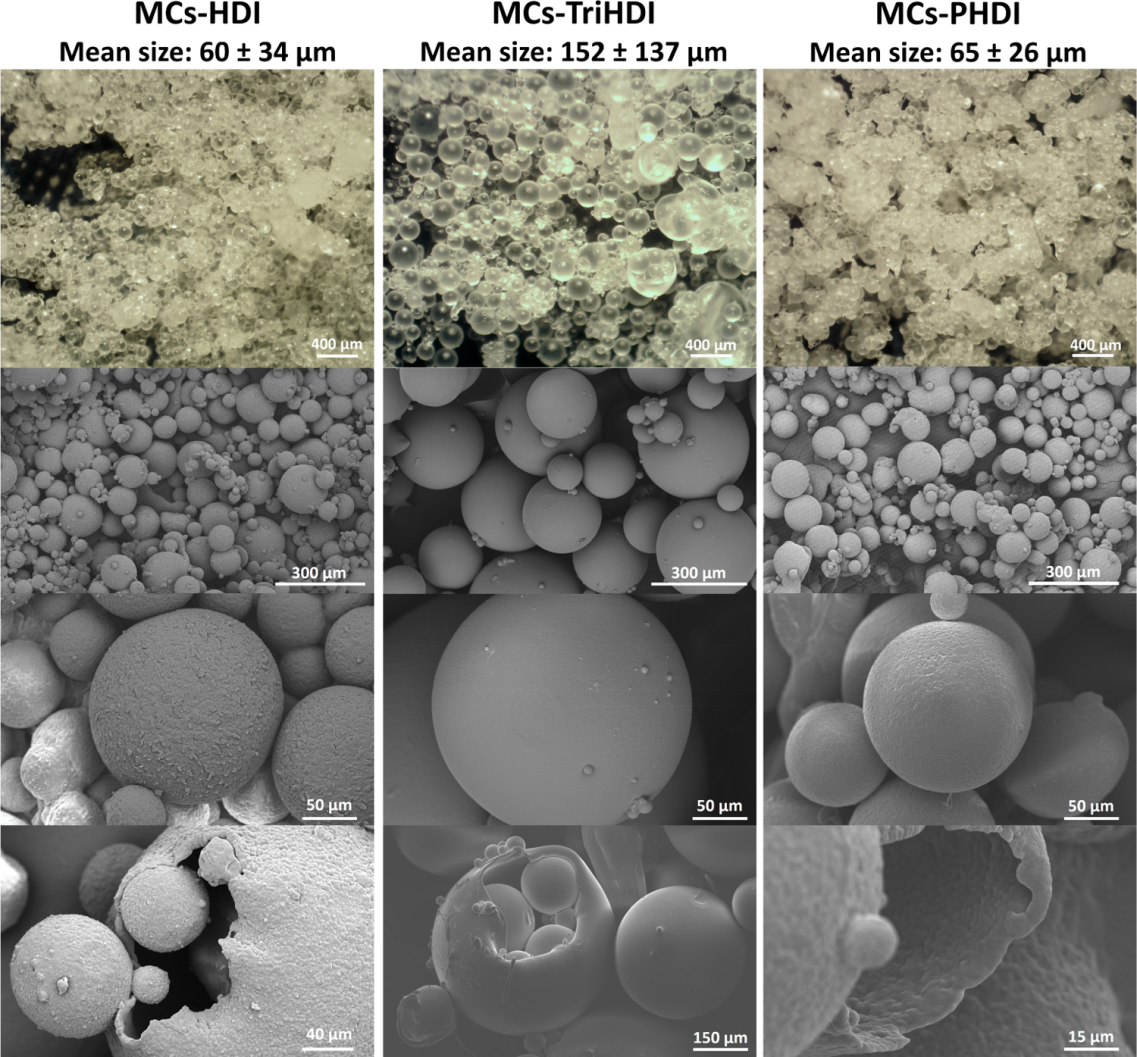
Optical microscopy photographs (first line) and SEM images of the MCs.

As depicted in Figure 2, the MCs exhibit a spherical shape without any discernible holes or cracks in the shell. The inherent characteristics of PBAT, including a glass transition temperature near −30 °C and Young’s modulus surpassing 400 MPa, contribute to the rubbery and tough nature of the MCs. The outer shell of MCs-HDI presents a coarse texture, while the surfaces of the other MCs, especially MCs-TriHDI, appear to be smoother. Analyses of the three types of MCs purposedly broken reveal core–shell structures featuring a thin shell (between 1.5 and 0.5 μm) with similar inner and outer surfaces. The absence of heterogeneities, spikes, and/or an external layer (Figure S2) indicates a successful encapsulation and protection of the isocyanates. MCs-HDI and MCs-PHDI exhibit loose MCs. Although there is some electrostatic attraction and surface forces between them, it is relatively easy to isolate the MCs. In contrast, MCs-TriHDI tend to aggregate and have surface adhesion points (Figure S3) that prevent the separation of the MCs.

The size distribution histograms (Figure S4) show right-skewed data, where the interquartile ranges (Q1–Q3) are 47, 142, and 40 μm, and the differences between the mean and median for MCs-HDI and MCs-PDI are less than 10 μm, whereas for MCs-TriHDI it is 50 μm. The increased size and size distribution of MCs-TriHDI are attributed to the much higher viscosity of TriHDI (6000 cP) compared to HDI (10 cP) or PHDI (1250 cP). The size of the MCs is influenced by the net shear stress available for droplet breakdown, and the viscous forces counteract the shear stresses in the organic phase. As the viscosity of the organic phase increases, the breakage of emulsion droplets becomes more challenging, leading to the formation of larger MCs and larger size distributions. It should be noted that the existence of larger MCs acting as containers for smaller MCs (Figures 2 and S5) can induce inaccuracies when analyzing the size and size distribution of MCs-TriHDI.

### Structural Characterization and Isocyanate Load

**Figure 3:**
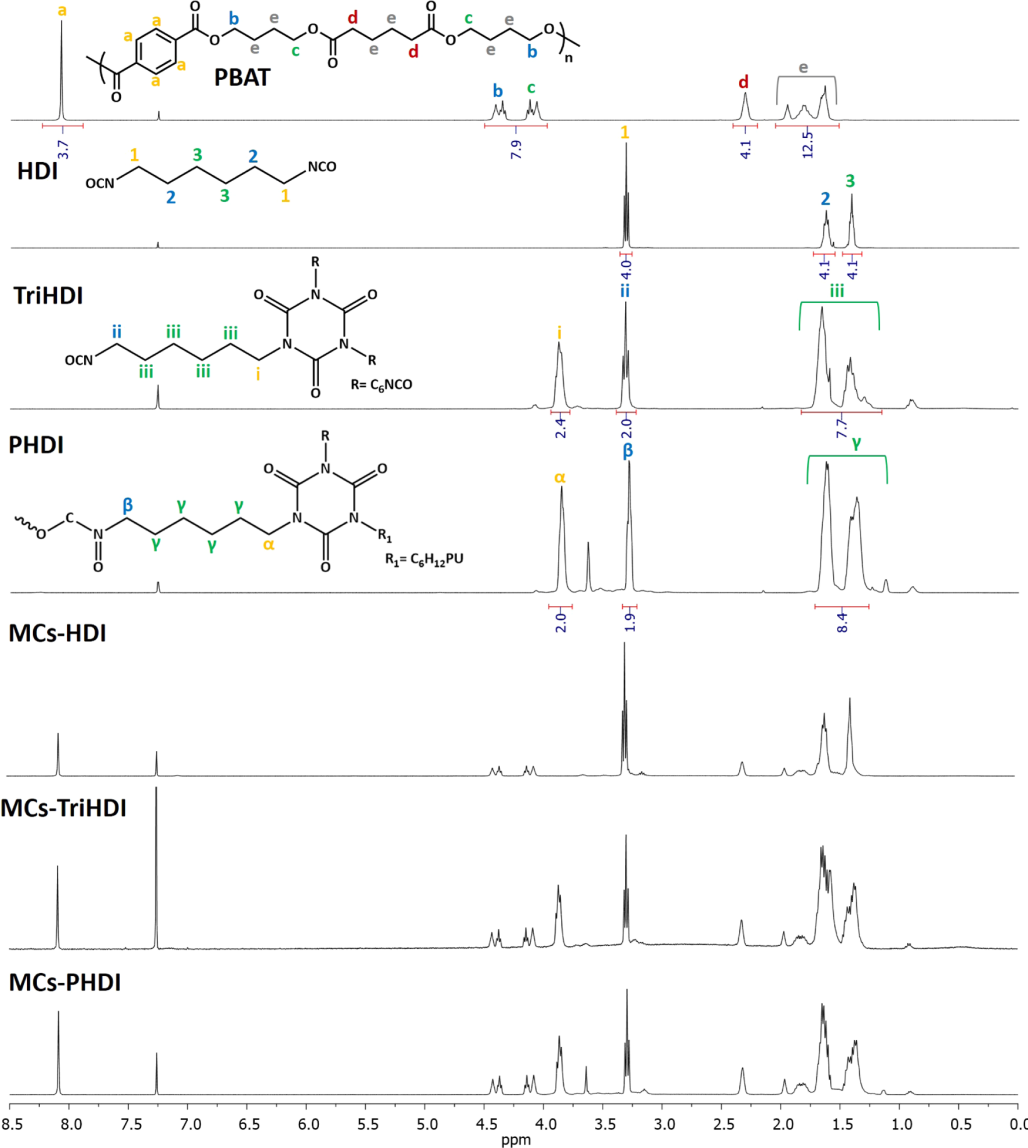
^1^H NMR spectra of the raw material and MCs in CDCl_3_.

The MCs were subjected to NMR, TGA, and Fourier-transform infrared spectroscopy (FTIR) analysis for qualitative and quantitative assessment. Figure 3 shows the ^1^H NMR spectra of the individual raw materials as well as the spectra of the MCs. The NMR signals were assigned based on their respective structures, and the ^1^H NMR spectra of the MCs exhibit a good merging with the spectra of the raw materials. The isocyanurate ring, in TriHDI and PHDI, causes the split of the triplet at 3.3 ppm, assigned to the –CH_2_– next to the isocyanate groups, into two signals (a triplet and a multiplet). The polyurea formation, by isocyanate reaction with water, affects the signals of the hydrogen protons, especially the protons directly correlated with the isocyanate group.^29^ Between 3 and 3.25 ppm, signals with very low intensity (magnification in Figure S6) appear, indicating a weak formation of polyurea and confirming that although it is not a 100% benign process for these isocyanates, the microencapsulation process is quite reliable.

**Figure 4:**
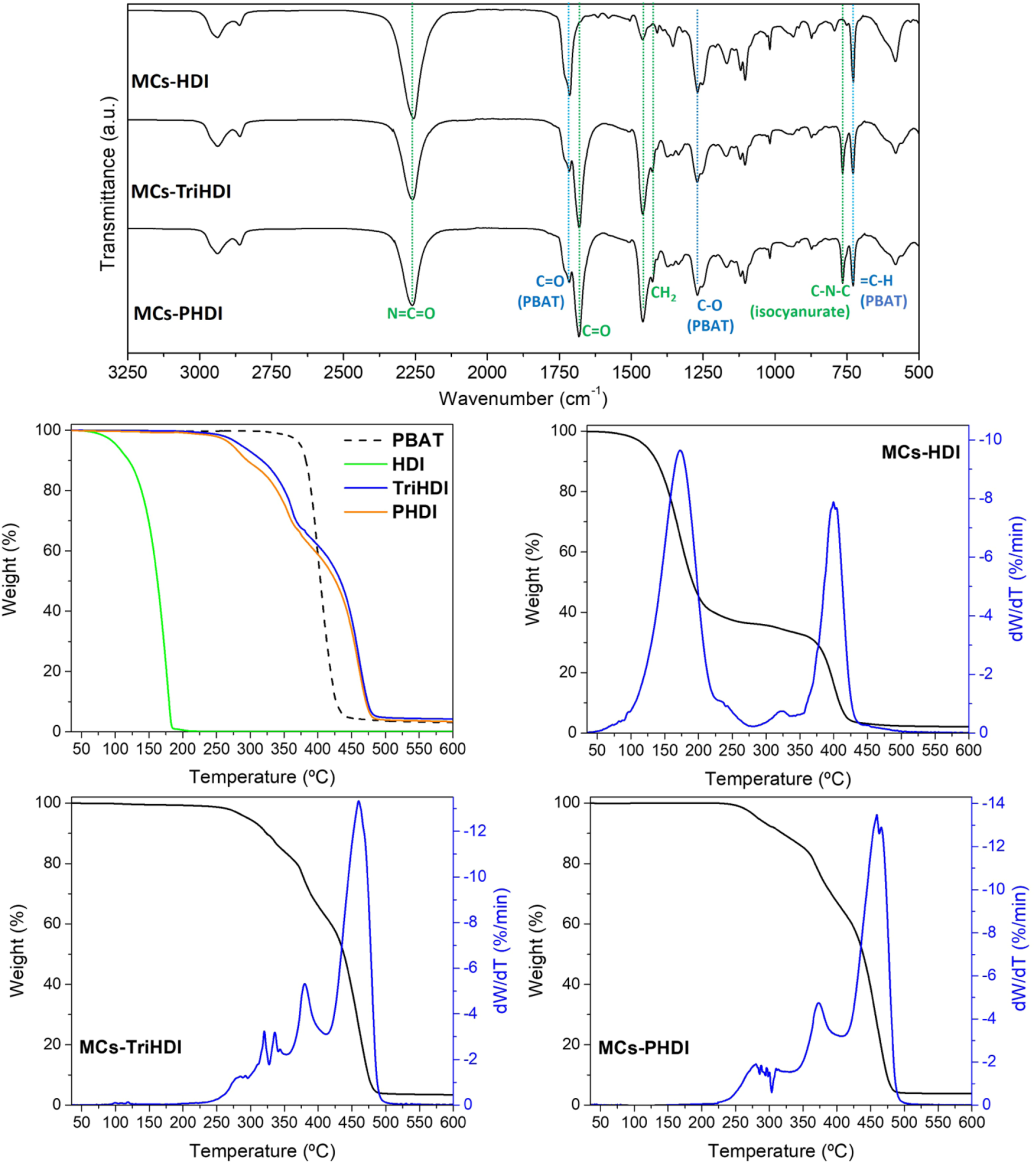
FTIR spectra of MCs (up). Thermograms of the raw material and MCs, obtained under a N_2_ atmosphere (down).

FTIR analysis was conducted to detect the isocyanate within the MCs and assess the shell’s composition by identifying characteristic vibrational groups. Figure 4 shows the FTIR spectra of the MCs, while Figure S7 shows the spectra of the raw materials. A high quantity of isocyanate can be confirmed by the intense band peak at ca. 2260 cm^–1^, attributed to N=C=O stretching vibrational frequency. The bands between 2830 and 3012 cm^–1^ are ascribed to C–H asymmetric and symmetric stretching vibrations from aliphatic and aromatic segments. The most prominent bands from PBAT are the carbonyl group (C=O) stretching vibrational band at 1714 cm^–1^, the stretching vibration of C–O–C at 1270 cm^–1^, and the sharp peak at 727 cm^–1^ from the out-plane =C–H (bending) in the benzene ring. The isocyanurate rings from TriHDI and PHDI are verified by the 1680 and 764 cm^–1^ bands arising from C=O stretching vibration at the heterocycle and the skeletal stretching vibration, respectively.^52^ CH_2_ asymmetric bending vibration can be found at 1460 cm^–1^ when attached only with carbons, at around 1426 cm^–1^ for those directly linked to the isocyanurate ring (only for TriHDI and PDI), and at 1356 cm^–1^ when attached to NCO groups.

As presented in Figures 4 and S8, the thermograms of HDI and PBAT show a single degradation event, with *T*_max_ value around 158 and 408 °C, respectively. TriHDI and PHDI have similar thermal characteristics, with the thermograms clearly showing three distinctive degradation phases observed within the temperature range of 191–515 °C, which have been previously documented for isocyanurates and polyurethanes.^53−55^ The presence of isocyanurate rings enhances the thermal stability of these compounds since the stage with the most significant weight loss displays a *T*_max_ value of approximately 460 °C.

The thermograms of the MCs demonstrate very good correspondence with the individual mass loss events of the isocyanate and PBAT components. In the case of HDI-MCs, a slight shift in the thermal event relative to HDI evaporation is observed, suggesting some protective effect provided by the polymeric shell. Additionally, an extra peak emerges at around 315 °C, representing approximately 3% of the total area and indicating a minor conversion of HDI into polyurea through a reaction with water during MC production. The MCs-HDI exhibit nearly complete degradation with a char yield of 2 wt % at 600 °C. In the case of MCs-TriHDI and MCs-PHDI, due to the multiple degradation steps occurring between 190 and 515 °C, it is not possible to isolate the PBAT degradation event in the thermograms. Consequently, a precise evaluation of the MCs’ payload and polyurea formation is complicated. Nonetheless, by comparing the peak ratios of the individual isocyanates with those of the MCs, it is possible to infer that little polyurea was formed.

Table 1 shows the isocyanate load of the developed MCs. Due to the overlapping of the degradation steps of PBAT with isocyanurate, it is not possible to determine the isocyanate weight percentage from MCs-TriHDI and MCs-PHDI by TGA. However, this evaluation was successfully conducted through titration and NMR analysis, either by direct comparison with a standard or by applying a calibration curve (Figures S1 and S9–S11). The isocyanate content in the MCs ranges between 63 and 68 wt % (NMR), and the results obtained through the three techniques are well-aligned. Considering the weight ratio of 3.25/6.80 g between the polymer and isocyanate, the load percentages are in line with the theoretical value of 68 wt % and demonstrate high encapsulation efficiencies. The differences are due to a small polyurea formation, resulting from the NCO groups’ reaction with water (from the emulsion or from atmospheric humidity). These encapsulation percentages are on par with the most notable reported results for isocyanate encapsulation.^56−61^

**Table 1:** Isocyanate Loading, Calculated by TGA, ^1^H NMR, Titration, and Their Follow-Up after 3 Months

MCs acronym	TGA (wt %)	NMR (wt %)	titration (wt %)	3 months (wt %)
MCs-HDI	63.7 ± 0.5	65.3 ± 1.2	61.0 ± 0.8	60.6 ± 0.7^a^
MCs-TriHDI	n/a	63.3 ± 0.5	64.3 ± 1.2	60.1 ± 1.7^b^
MCs-PHDI	n/a	67.7 ± 1.2	65.3 ± 1.7	66.5 ± 0.5^b^

a Obtained by TGA.

b Obtained by NMR.

Three months later, the MCs were analyzed, and a reduction of less than 5% of their initial isocyanate payload was observed. A small polyurea formation can be assessed by TGA or FTIR (Figures S12 and S13). In the thermograms, the widening of the HDI degradation step (50–275 °C) after 3 months, as well as the increase in weight loss at 300–350 and 425–475 °C, is an indication of the onset of monomer polymerization and the formation of some polyurea moieties.^29^ In the FTIR spectra, the small fraction of formed polyurea can also be noticed by a slight increase in the bands attributed to N–H stretching and bending (3341 and 1500–1600 cm^–1^). It should be stressed that the NCO stretch vibration bond is still very prominent, which evidences that encapsulation effectively protects the isocyanate species.

The permeability of the PBAT shell was tested under the influence of ethyl acetate, toluene, and hexane. These solvents are quite common in commercially available adhesives and are present in the commercial adhesive used in this work (Plastik 6275). For this study, we used 0.1 g of MCs dispersed in 5 mL of solvent, and after 1 and 3 days, we analyzed the MCs by TGA. As shown by the thermograms in Figure S14, the three solvents leached the isocyanates. For example, in the case of MCs-HDI, there is a decrease between 85 and 90% of the initial isocyanate load, after 1 day. It is noteworthy that the MCs sustain their shape after being placed in the solvents and do not show fissures (Figure S14). These results allow us to conclude that the PBAT MCs, after some time mixed with the adhesive, may release isocyanate and start the curing process. Therefore, PBAT MCs can only be used in two-component adhesives, meaning they must be supplied separately from the adhesive and mixed only at the time of bonding.

### Adhesive Formulation and Adhesive Joint Characterization

The adhesive formulations were prepared by mixing either MCs or nonencapsulated isocyanates with a polyurethane-based adhesive (Plastik 6275). Considering previous results using IPDI and Plastik 6275,^62^ a formulation containing 5 wt % isocyanate was prepared.

**Figure 5:**
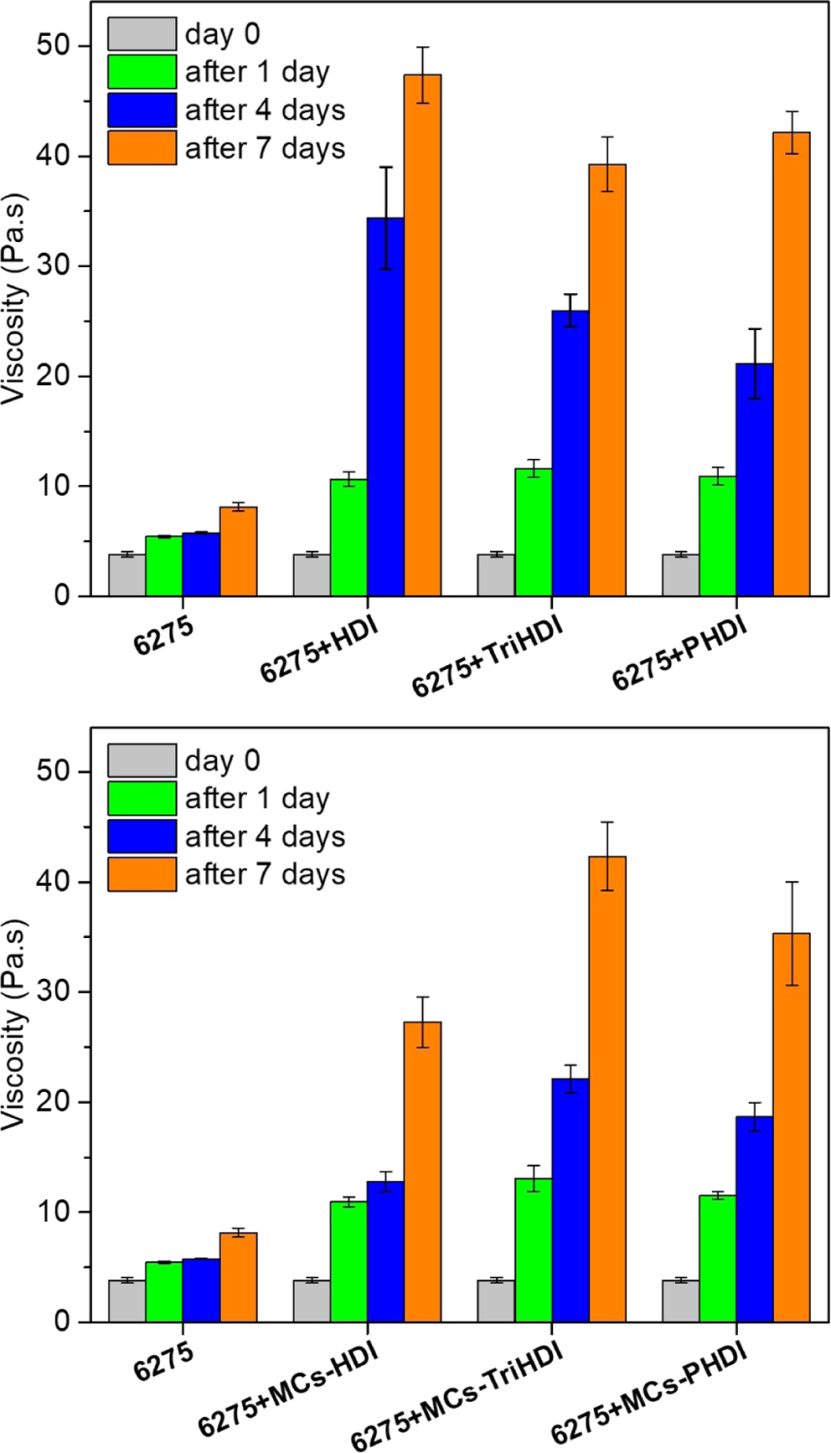
Viscosity of the adhesive formulations with nonencapsulated isocyanates (up) and encapsulated isocyanates (down), over 1 week.

To characterize the cross-linking capability of different isocyanates and the microencapsulated isocyanates, we studied the viscosity of different formulations over 7 days. After the mixing of the adhesive and isocyanate, the open time of the adhesive decreases, and the curing speed increases considerably since the isocyanate reacts with the free hydroxyl groups of the adhesive and water present in the air, forming polyurethane and polyurea bonds. As a result, the viscosity of the solution will increase. Figure 5 represents the evolution of the viscosity values of the formulations over 7 days of mixing (data in Table S3) and clearly shows the influence of the isocyanates.

The formulation using only Plastik 6275 showed a 42% increase in viscosity after 1 day (from 3.84 to 5.45 Pa·s) and a 112% increase after 1 week (from 3.84 to 8.15 Pa·s). Formulations incorporating isocyanates displayed a viscosity increase of at least 178% after 1 day, reaching up to 1133% after a week. It was also observed that significant cross-linking had already occurred on the fourth day, with HDI leading to faster curing. This can be attributed to its lower molecular weight and a higher percentage of NCO per molecule (49.7% compared to 21–23% for TriHDI and PHDI). By comparing the results obtained with nonencapsulated and encapsulated isocyanates, we concluded that MCs provide some protection to the isocyanate, even considering that the PBAT shell is permeable to solvents in Plastik 6275. The effect of MCs is particularly noticeable for HDI as the viscosity after 7 days rose to only 27.2 Pa·s, in contrast to the 47.4 Pa·s obtained with nonencapsulated HDI.

The MCs-HDI and MCs-PHDI exhibit smaller dimensions and allow for a good dispersion throughout the adhesive formulation and an effective distribution on the substrate. Conversely, the MCs-TriHDI possess larger sizes and show a higher degree of aggregation, resulting in the formation of lumps and poorer homogenization on the adhesive joint film (Figure S16).

**Figure 6:**
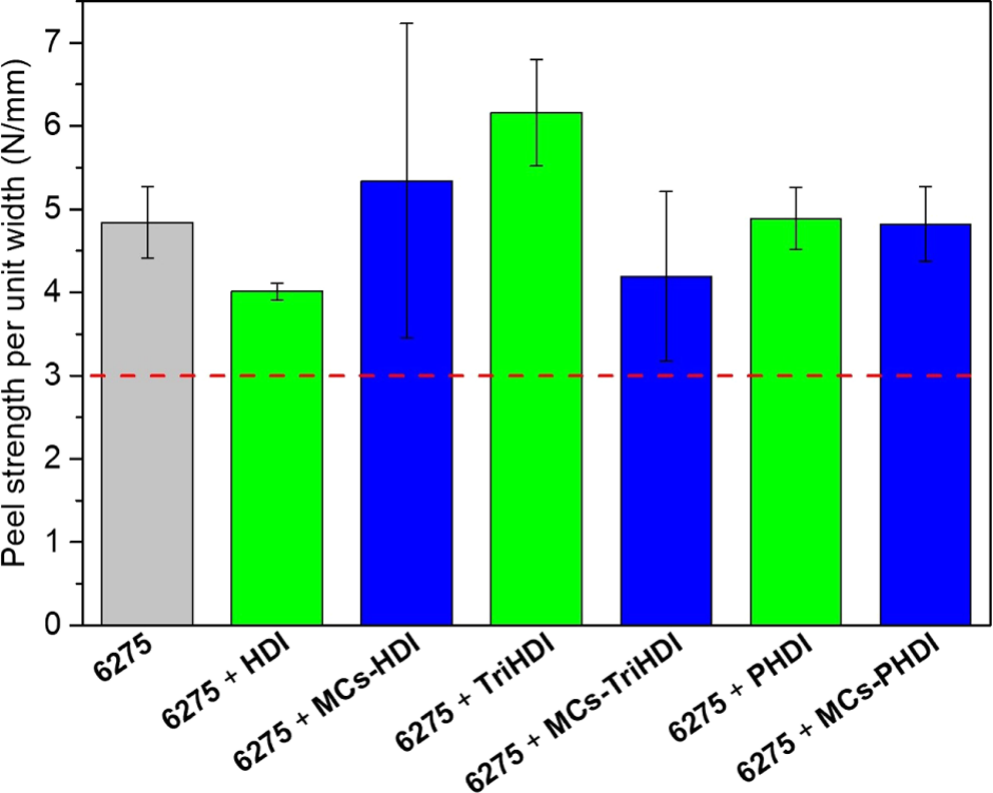
Results of the peel tests (average of three adhesive joints).

In the footwear industry, adhesive bonds must meet specific standards to ensure the shoe’s durability. For instance, the required peel strength values for joining the upper and sole of casual footwear should be higher than 3 N/mm, while mountain footwear should be higher than 3.5 N/mm.^63^ Peel strength represents the required mechanical force per unit width to detach two bonded materials. Its analysis was conducted at a constant speed rate of 100 mm/min, and the force exerted during the test was divided by the substrate’s width (30 mm) to obtain the peel strength value. Figure 6 depicts the average values of the peel strength per width of different tested adhesive joints, and the data are presented in Table S4.

As the isocyanate-free formulation (Plastik 6275) already demonstrates a high peel strength, the peeling test was not conducted to show the exceptional adhesive strength of the prepared formulations. Instead, it was conducted to ascertain whether the MCs’ polymeric walls would damage the adhesive joint’s quality by potentially reducing the bond strength.

The adhesives with nonencapsulated HDI exhibit a slight reduction in peel strength, while the other adhesives either demonstrate a slight increase or remain roughly at the same level (considering the standard deviation). This difference in HDI adhesives could be explained by the high volatility of HDI, which causes isocyanate to be lost during the preparation of the adhesive joint, specifically during the drying and reactivation step. The fact that MCs-TriHDI create lumps and a not-so-homogeneous distribution in the adhesive film explains the worse results obtained with MCs-TriHDI compared to nonencapsulated isocyanate (TriHDI).

After the peel test, the substrates exhibited a complete rupture of the MCs, which confirmed a good isocyanate release during adhesive joint manufacturing. All of the samples have shown peel strength values above 3 N/mm, which validates the use of these MCs for footwear adhesives. Additionally, all of the peel tests induced substrate failure, or substrate failure combined with small cohesion failure (Figure S17), which means that the adhesive bond exceeds the strength of the substrate itself, at least in some points of the bond, proving once more the high bonding performance.

The creep test enables us to gauge the temperature endurance of an adhesive under a constant stress without compromising its structure. This attribute holds particular significance given that footwear may be exposed to a wide range of temperatures throughout transportation, exposure, or utilization. The creep test was conducted under controlled conditions, starting at 60 °C.

The creep test results (Table 2) confirm that adding 5 wt % isocyanate significantly enhances the temperature resistance of the adhesive bond. While the inclusion of nonencapsulated HDI improved thermal resistance to some extent, the joints still experienced detachment across all temperature ranges. However, with MCs-HDI, the detachment started only at 80 °C, indicating that encapsulation protects the isocyanate from premature evaporation, leading to improved outcomes. TriHDI adhesives showed a slight opening at 70 °C, yet demonstrated better resistance at 90 °C than HDI adhesives. Conversely, MCs-TriHDI adhesives exhibited slightly worse results due to a less effective distribution within the adhesive film. Even so, specimens with MCs-TriHDI displayed an opening of only 3.1 cm at 90 °C. PHDI and MCs-PHDI adhesive joints displayed outstanding performance as the bonded substrates showed no opening in any temperature range. Therefore, MCs-PHDI are the optimal choice for temperature-resistance adhesive joints. Moreover, it is noteworthy to highlight that the creep test results obtained from PBAT microcapsules and HDI derivatives, revealed in this work, far surpass those reported for other polymers with isocyanate encapsulated,^62^ and they stand as the best options considering the similar systems published so far.

**Table 2:** Results of Creep Tests^a^

sample	60 °C	70 °C	80 °C	90 °C
6275^b^	1.0 ± 0.3 cm	full opening	full opening	full opening
6275 + HDI	0.33 ± 0.05 cm	1.2 ± 0.7 cm	9.3 ± 1.0 cm	full opening
6275 + MCs-HDI	0 ± 0 cm	0 ± 0 cm	0.77 ± 0.25 cm	full opening
6275 + TriHDI	0 ± 0 cm	0.27 ± 0.05 cm	0.63 ± 0.12 cm	2.8 ± 1.0 cm
6275 + MCs-TriHDI	0.07 ± 0.09 cm	0.57 ± 0.17 cm	1.7 ± 0.9 cm	3.1 ± 0.8 cm
6275 + PHDI	0 ± 0 cm	0 ± 0 cm	0 ± 0 cm	0 ± 0 cm
6275 + MCs-PHDI	0 ± 0 cm	0 ± 0 cm	0 ± 0 cm	0 ± 0 cm

a The values indicate the extent of substrate detachment. A full opening corresponds to a minimum detachment of 10 cm.

b Previous work.^62^

### Environmental Assessment Results

Through an environmental assessment methodology, the potential environmental effects of the commercial adhesive formulation (CA), composed of Plastik 6275 and nonencapsulated isocyanate (TDI derivative), and the formulation with Plastik 6275 and MCs-PHDI (MCA) were evaluated. The MCs-PHDI were chosen based on the excellent peel and creep test results. To further simulate production in the industrial context, the environmental assessment used the outcomes from the closed-system approach for MC production, which involves solvent recovery.

For both systems (CA and MCA), the results indicate that the primary factor contributing to the environmental impact, in terms of global warming potential (GWP) and energy consumption (EC) indicators, is the production stage (Table S5). Within the production stage, acetone usage plays a very significant role in the environmental impact of the CA system (Figure S18). In the MCA system, acetone consumption remains the most significant contributor, but MC production also contributes to GWP (27%) and EC (19%), as shown in Figure S19.

**Figure 7:**
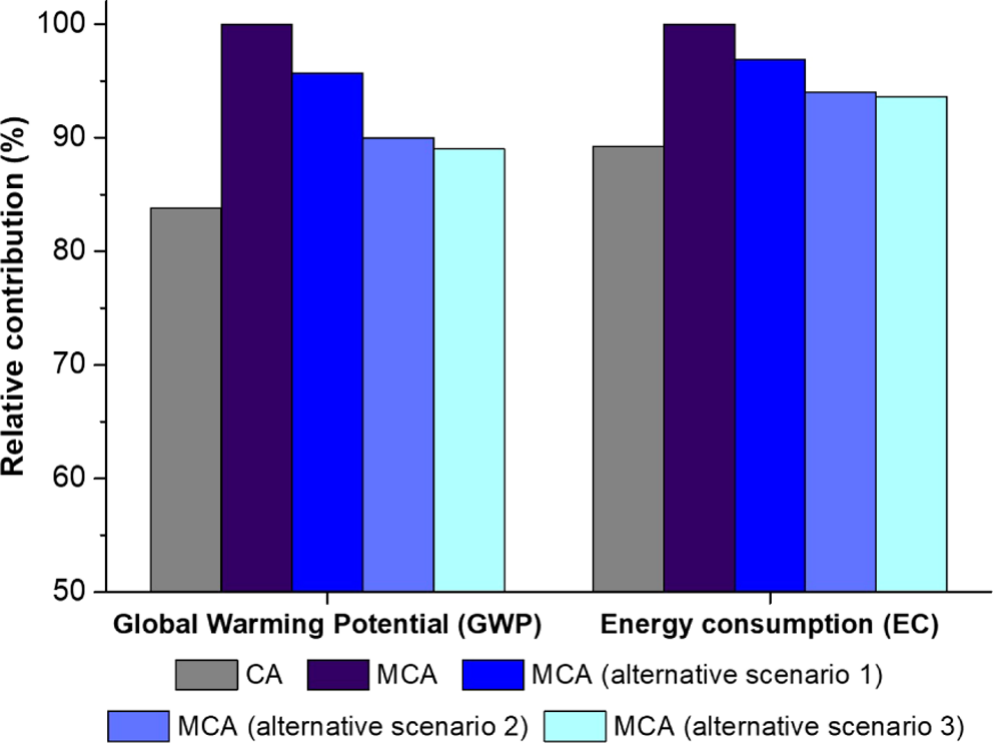
Comparison of relative contributions for GWP and EC of the CA and MCA systems and the three alternative scenarios of the MCA system.

Due to the additional steps associated with MC production, the MCA system exhibits higher values in both GWP and EC. As depicted in Figure 7, the MCA system shows a 16.2% increase in GWP and a 10.8% increase in EC.

Multiple scenarios were explored as the environmental assessment was carried out before the actual industrial production of MCs, and certain production parameters can be fine-tuned on an industrial scale. Three cumulative alternative realistic scenarios for the MCA systems were evaluated: (i) alternative scenario 1, involves enhancing MC production efficiency from 70 to 90%; (ii) alternative scenario 2, built upon scenario 1 by improving the DCM recovery efficiency during MC production from 65 to 90%; (iii) alternative scenario 3 combines scenarios 1 and 2 while also considering an alternative energy source for the MCA using photovoltaic panels instead of the electricity mix. Figure 7 shows the comparative outcomes among the CA and MCA systems along with the alternative scenarios. As expected, the three alternative scenarios exhibit reduced environmental impacts compared to the standard MCA. This effect could be further enhanced if the recovered solvent was reused to produce MCs. Additionally, the results indicate that adopting an alternative renewable energy source (alternative scenario 3) does not generate a substantial reduction in the environmental indicators as the optimization of yield production and solvent recovery (alternative scenarios 1 and 2). The disparities between the outcomes of the alternative scenarios and the CA system fall well below 10%, except for alternative scenario 1 in GWP. Assuming that deviations of up to 10% guided by model, scenario, and parameter uncertainties are deemed irrelevant,^64^ the alternative MCA scenarios can be viewed as comparable to the CA system.

## Conclusions

This study represents the first attempt at encapsulating HDI derivatives through a straightforward method based on solvent evaporation and using PBAT as the shell material. It further reports an expressive recovery of the organic solvent (65% after 90 min), which can be reused in the production of MCs, strongly contributing to the sustainability of this process. Three HDI derivatives, monomer (HDI), trimer (TriHDI), and polymer (PHDI), were successfully encapsulated by a thin layer of PBAT, yielding MCs with spherical core–shell structures. The MCs possess smooth and intact thin shells that are free from holes or cracks. MCs-HDI and MCs-PHDI display mean diameters of 60 and 65 μm, respectively. In contrast, MCs-TriHDI stands out with a larger average diameter of 152 μm and aggregates. The MCs were obtained with an isocyanate payload exceeding 60 wt %, with MCs-PHDI reaching up to 68 wt %. The small amount of polyurea detected immediately after the production and after 3 months demonstrates the effectiveness of the PBAT shell protection and the MCs’ acceptable shelf-life for this application.

The incorporation of MCs-HDI and MCs-PHDI into the adhesive formulation led to their effective dispersion, facilitating a uniform spread within the substrates. As intended, all MCs release isocyanates during the bonding process. The peel strength values, ranging from 4.19 to 5.34 N/mm, are significantly higher than the threshold required for the high-quality footwear industry (≥3 N/mm) and induced substrate failure, or substrate failure combined with small cohesion failure. Therefore, we can conclude that the PBAT shell does not negatively influence the peel strength of the adhesive joint, and the results are consistent with those required in the footwear industry. The creep tests confirm that 5 wt % isocyanate significantly enhances the temperature resistance of the adhesive joint. The comparison between encapsulated and nonencapsulated HDI shows that MCs-HDI perform substantially better, even though both lead to more than 10 cm displacement at 90 °C. The MCs-PHDI demonstrate outstanding performance as the substrates show no opening, even at 90 °C. Considering all of the results, we can conclude that the MCs-PHDI contribute to the best adhesive formulations in this work and the literature.

The environmental assessment demonstrates that the formulation with MCs-PHDI shows higher GWP and EC environmental indicators in comparison to commercial adhesive formulations. This is due to the supplementary steps linked to the production of MCs. The study of realistic alternative scenarios with readily applicable features in an industrial setting shows that the differences between the two formulation systems can be negligible.

In summary, our research introduces a straightforward approach to manufacturing MCs loaded with a high amount of isocyanates with the potential for application in a pilot-scale setup. The microencapsulation enables safer handling of toxic isocyanate species and provides enhanced protection for isocyanates. Incorporating MCs loaded with isocyanates as hardeners in adhesives has led to strong adhesive joints with creep resistance up to 90 °C. This innovative adhesive can be considered equivalent to commercial adhesive formulations in terms of their performance and environmental impact. Additionally, it offers the advantage of avoiding the direct handling of toxic isocyanate species and complies with the recent REACH regulation on isocyanates.

## Supporting Information Available

NMR, FTIR, SEM, and TGA data from MCs’ characterization; photographs of the adhesive formulation and adhesive joints; peel test data; and additional information regarding the environmental assessment (PDF)

## References

[ref1] Eram S.; Fahmina Z. Polyurethane: An Introduction. In Polyurethane; Fahmina Z.; Eram S., Eds.; IntechOpen: Rijeka, 2012; pp 1–14.

[ref2] Michael S. Structure–Property Relations in Polyurethanes. In Szycher’s Handbook of Polyurethanes; CRC Press, 2012; pp 87–135.

[ref3] Wright H. C.; Cameron D. D.; Ryan A. J. Rational Design of a Polyurethane Foam. Polymers 2022, 14 (23), 5111 10.3390/polym14235111. 36501508 PMC9736621

[ref4] Gonçalves D.; Bordado J. M.; Marques A. C.; Galhano dos Santos R. Non-Formaldehyde, Bio-Based Adhesives for Use in Wood-Based Panel Manufacturing Industry—A Review. Polymers 2021, 13 (23), 4086 10.3390/polym13234086. 34883590 PMC8658755

[ref5] Poljanšek I.; Fabjan E.; Moderc D.; Kukanja D. The effect of free isocyanate content on properties of one component urethane adhesive. Int. J. Adhes. Adhes. 2014, 51, 87–94. 10.1016/j.ijadhadh.2014.02.012.

[ref6] Attaei M.; Calado L. M.; Morozov Y.; Taryba M. G.; Shakoor R. A.; Kahraman R.; Marques A. C.; Montemor M. F. Smart epoxy coating modified with isophorone diisocyanate microcapsules and cerium organophosphate for multilevel corrosion protection of carbon steel. Prog. Org. Coat. 2020, 147, 105864 10.1016/j.porgcoat.2020.105864.

[ref7] Engels H. W.; Pirkl H. G.; Albers R.; Albach R. W.; Krause J.; Hoffmann A.; Casselmann H.; Dormish J. Polyurethanes: versatile materials and sustainable problem solvers for today’s challenges. Angew. Chem., Int. Ed. 2013, 52 (36), 9422–9441. 10.1002/anie.201302766. 23893938

[ref8] Rolph M. S.; Markowska A. L. J.; Warriner C. N.; O’Reilly R. K. Blocked isocyanates: from analytical and experimental considerations to non-polyurethane applications. Polym. Chem. 2016, 7 (48), 7351–7364. 10.1039/C6PY01776B.

[ref9] Naldzhiev D.; Mumovic D.; Strlic M. Polyurethane insulation and household products—A systematic review of their impact on indoor environmental quality. Build. Sci. 2020, 169, 106559 10.1016/j.buildenv.2019.106559.

[ref10] Speciality Plastics in Cardiovascular Applications. In Plastics in Medical Devices for Cardiovascular Applications; Padsalgikar A. D., Ed.; William Andrew Publishing, 2017; Chapter 3, pp 53–82.

[ref11] Martín-Martínez J. M. Shoe Industry. In Handbook of Adhesion Technology; da Silva L. F. M.; Öchsner A.; Adams R. D., Eds.; Springer: Berlin, Heidelberg, 2011; pp 1315–1347.

[ref12] Paiva R. M.; Marques E. A.; da Silva L. F.; António C. A.; Arán-Ais F. Adhesives in the footwear industry. Proc. Inst. Mech. Eng., Part L 2016, 230 (2), 357–374. 10.1177/1464420715602441.

[ref13] Lockey J. E.; Redlich C. A.; Streicher R.; Pfahles-Hutchens A.; Hakkinen P. B.; Ellison G. L.; Harber P.; Utell M.; Holland J.; Comai A.; White M. Isocyanates and human health: multistakeholder information needs and research priorities. J. Occup. Environ. Med. 2015, 57 (1), 44–51. 10.1097/JOM.0000000000000278. 25563538 PMC4286799

[ref14] Bello D.; Woskie S. R.; Streicher R. P.; Liu Y.; Stowe M. H.; Eisen E. A.; Ellenbecker M. J.; Sparer J.; Youngs F.; Cullen M. R.; Redlich C. A. Polyisocyanates in occupational environments: A critical review of exposure limits and metrics. Am. J. Ind. Med. 2004, 46 (5), 480–491. 10.1002/ajim.20076. 15490474

[ref15] Commission Regulation (EU). Official Journal of the European Union, 2020. https://eurlex.europa.eu/eli/reg/2020/1149/oj.

[ref16] Calderón-Oliver M.; Ponce-Alquicira E. The Role of Microencapsulation in Food Application. Molecules 2022, 27 (5), 1–16. 10.3390/molecules27051499. PMC8912024 35268603

[ref17] Saifullah M.; Shishir M. R. I.; Ferdowsi R.; Tanver Rahman M. R.; Van Vuong Q. Micro and nano encapsulation, retention and controlled release of flavor and aroma compounds: A critical review. Trends Food Sci. Technol. 2019, 86, 230–251. 10.1016/j.tifs.2019.02.030.

[ref18] Lengyel M.; Kállai-Szabó N.; Antal V.; Laki A. J.; Antal I. Microparticles, Microspheres, and Microcapsules for Advanced Drug Delivery. Sci. Pharm. 2019, 87 (3), 20 10.3390/scipharm87030020.

[ref19] Zhao M.; Chen Z.; Hao L.; Chen H.; Zhou X.; Zhou H. CMC based microcapsules for smart delivery of pesticides with reduced risks to the environment. Carbohydr. Polym. 2023, 300, 120260 10.1016/j.carbpol.2022.120260. 36372488

[ref20] Wang R.; Liu S.; Sun F.-s.; Yu X.; Liu X.; Li B.-x.; Mu W.; Zhang D.-x.; Liu F. Balance the rapid release of insecticide microcapsules using double-layer shielding effect when the foliar application. Chem. Eng. J. 2023, 455, 140899 10.1016/j.cej.2022.140899.

[ref21] Chen Y.; Li W.; Luo J.; Liu R.; Sun G.; Liu X. Robust Damage-Reporting Strategy Enabled by Dual-Compartment Microcapsules. ACS Appl. Mater. Interfaces 2021, 13 (12), 14518–14529. 10.1021/acsami.0c20276. 33739100

[ref22] Liu S.; Xie H.; Cao Q.; Ning Y.; Song Y.; Zhang C.; Liu B. Preparation of a novel IPDI/PUF@CeO2 bi-functional microcapsules and its improvement for the self-healing and anti-corrosion performance in epoxy coatings. Prog. Org. Coat. 2022, 169, 106897 10.1016/j.porgcoat.2022.106897.

[ref23] Wu K.; Chen Y.; Luo J.; Liu R.; Sun G.; Liu X. Preparation of dual-chamber microcapsule by Pickering emulsion for self-healing application with ultra-high healing efficiency. J. Colloid Interface Sci. 2021, 600, 660–669. 10.1016/j.jcis.2021.05.066. 34049021

[ref24] Attaei M.; Loureiro M. V.; Do Vale M.; Condeço J. A. D.; Pinho I.; Bordado J. C.; Marques A. C. Isophorone Diisocyanate (IPDI) Microencapsulation for Mono-Component Adhesives: Effect of the Active H and NCO Sources. Polymers 2018, 10 (8), 825 10.3390/polym10080825. 30960750 PMC6403942

[ref25] Arán-Ais F.; Pérez-Limiñana M. Á.; Sánchez-Navarro M. M.; Orgilés-Barceló C. Developments in Microencapsulation Technology to Improve Adhesive Formulations. J. Adhes. 2012, 88 (4–6), 391–405. 10.1080/00218464.2012.660368.

[ref26] Messersmith R. E.; Bartlett M. E.; Rose D. J.; Smith D. A.; Patchan M. W.; Benkoski J. J.; Trexler M. M.; Hoffman C. M. Rapid Underwater Adhesive Utilizing Crosslinker and Amine Catalyst-Filled Microcapsules. ACS Appl. Polym. Mater. 2021, 3 (2), 996–1002. 10.1021/acsapm.0c01277.

[ref27] Perignon C.; Ongmayeb G.; Neufeld R.; Frere Y.; Poncelet D. Microencapsulation by interfacial polymerisation: membrane formation and structure. J. Microencapsulation 2015, 32 (1), 1–15. 10.3109/02652048.2014.950711. 25265057

[ref28] Loureiro M. V.; Attaei M.; Rocha S.; Vale M.; Bordado J. C.; Simões R.; Pinho I.; Marques A. C. The role played by different active hydrogen sources in the microencapsulation of a commercial oligomeric diisocyanate. J. Mater. Sci. 2020, 55 (11), 4607–4623. 10.1007/s10853-019-04301-1.

[ref29] Aguiar A.; Mariquito A.; Gonçalves D.; Pinho I.; Marques A. C. Biodegradable Microcapsules of Poly(Butylene Adipate-co-Terephthalate) (PBAT) as Isocyanate Carriers and the Effect of the Process Parameters. Polymers 2023, 15 (3), 665 10.3390/polym15030665. 36771965 PMC9921966

[ref30] Delgado B.; Carrêlo H.; Loureiro M. V.; Marques A. C.; Borges J. P.; Cidade M. T. Injectable hydrogels with two different rates of drug release based on pluronic/water system filled with poly(ε-caprolactone) microcapsules. J. Mater. Sci. 2021, 56 (23), 13416–13428. 10.1007/s10853-021-06156-x.

[ref31] Chaiyasat P.; Pholsrimuang P.; Boontung W.; Chaiyasat A. Influence of Poly(L-lactic acid) Molecular Weight on the Encapsulation Efficiency of Urea in Microcapsule Using a Simple Solvent Evaporation Technique. Polym.-Plast. Technol. Eng. 2016, 55 (11), 1131–1136. 10.1080/03602559.2015.1132447.

[ref32] Brunner C. T.; Baran E. T.; Pinho E. D.; Reis R. L.; Neves N. M. Performance of biodegradable microcapsules of poly(butylene succinate), poly(butylene succinate-co-adipate) and poly(butylene terephthalate-co-adipate) as drug encapsulation systems. Colloids Surf., B 2011, 84 (2), 498–507. 10.1016/j.colsurfb.2011.02.005. 21376545

[ref33] Park J. H.; Ye M.; Park K. Biodegradable polymers for microencapsulation of drugs. Molecules 2005, 10 (1), 146–161. 10.3390/10010146. 18007283 PMC6147704

[ref34] Ibraheem D.; Iqbal M.; Agusti G.; Fessi H.; Elaissari A. Effects of process parameters on the colloidal properties of polycaprolactone microparticles prepared by double emulsion like process. Colloids Surf., A 2014, 445, 79–91. 10.1016/j.colsurfa.2014.01.012.

[ref35] Costa A.; Encarnação T.; Tavares R.; Todo Bom T.; Mateus A. Bioplastics: Innovation for Green Transition. Polymers 2023, 15 (3), 517 10.3390/polym15030517. 36771817 PMC9920607

[ref36] Loureiro M. V.; Vale M.; Galhano R.; Matos S.; Bordado J. C.; Pinho I.; Marques A. C. Microencapsulation of Isocyanate in Biodegradable Poly(ε-caprolactone) Capsules and Application in Monocomponent Green Adhesives. ACS Appl. Polym. Mater. 2020, 2 (11), 4425–4438. 10.1021/acsapm.0c00535.

[ref37] Barbosa R. F. d. S.; Yudice E. D. C.; Mitra S. K.; Rosa D. d. S. Characterization of Rosewood and Cinnamon Cassia essential oil polymeric capsules: Stability, loading efficiency, release rate and antimicrobial properties. Food Control 2021, 121, 107605 10.1016/j.foodcont.2020.107605.

[ref38] Schindelin J.; Arganda-Carreras I.; Frise E.; Kaynig V.; Longair M.; Pietzsch T.; Preibisch S.; Rueden C.; Saalfeld S.; Schmid B.; Tinevez J.-Y.; White D. J.; Hartenstein V.; Eliceiri K.; Tomancak P.; Cardona A. Fiji: an open-source platform for biological-image analysis. Nat. Methods 2012, 9 (7), 676–682. 10.1038/nmeth.2019. 22743772 PMC3855844

[ref39] ASTM D2572-19. Standard Test Method for Isocyanate Groups in Urethane Materials or Prepolymers; American Society for Testing and Materials, 2019; Vol. 2.

[ref40] Bharti S. K.; Roy R. Quantitative 1H NMR spectroscopy. TrAC, Trends Anal. Chem. 2012, 35, 5–26. 10.1016/j.trac.2012.02.007.

[ref41] Finnveden G.; Hauschild M. Z.; Ekvall T.; Guinée J.; Heijungs R.; Hellweg S.; Koehler A.; Pennington D.; Suh S. Recent developments in Life Cycle Assessment. J. Environ. Manage. 2009, 91 (1), 1–21. 10.1016/j.jenvman.2009.06.018. 19716647

[ref42] Pennington D. W.; Potting J.; Finnveden G.; Lindeijer E.; Jolliet O.; Rydberg T.; Rebitzer G. Life cycle assessment Part 2: Current impact assessment practice. Environ. Int. 2004, 30 (5), 721–739. 10.1016/j.envint.2003.12.009. 15051247

[ref43] Rebitzer G.; Ekvall T.; Frischknecht R.; Hunkeler D.; Norris G.; Rydberg T.; Schmidt W. P.; Suh S.; Weidema B. P.; Pennington D. W. Life cycle assessment part 1: framework, goal and scope definition, inventory analysis, and applications. Environ. Int. 2004, 30 (5), 701–720. 10.1016/j.envint.2003.11.005. 15051246

[ref44] International Organization for Standardization. Environmental Management e Life Cycle Assessment e Principles and Framework; ISO: Geneve, Switzerland, 2006.

[ref45] Goedkoop M.; Oele M.; Effting S. SimaPro Database Manual: Methods Library; PRe consultants B.V.: The Netherlands, 2004.

[ref46] Capello C.; Wernet G.; Sutter J.; Hellweg S.; Hungerbühler K. A comprehensive environmental assessment of petrochemical solvent production. Int. J. Life Cycle Assess. 2009, 14 (5), 467–479. 10.1007/s11367-009-0094-4.

[ref47] Huijbregts M. A. J.; Rombouts L. J. A.; Hellweg S.; Frischknecht R.; Hendriks A. J.; van de Meent D.; Ragas A. M. J.; Reijnders L.; Struijs J. Is Cumulative Fossil Energy Demand a Useful Indicator for the Environmental Performance of Products?. Environ. Sci. Technol. 2006, 40 (3), 641–648. 10.1021/es051689g. 16509298

[ref48] The Swiss Centre for Life Cycle Inventories, ecoinvent database version 3.4; Zürich, Switzerland, 2017.

[ref49] European Life Cycle Database (ELCD) v3.2 Database; European Commission: Brussels, Belgium, 2017.

[ref50] PRé V. A. SimaPro Database Manual, Methods Library, report version 3.0; PRé Consultants: Netherlands, 2017.

[ref51] Li M.; Rouaud O.; Poncelet D. Microencapsulation by solvent evaporation: State of the art for process engineering approaches. Int. J. Pharm. 2008, 363 (1), 26–39. 10.1016/j.ijpharm.2008.07.018. 18706988

[ref52] Hu J.; Chen Z.; He Y.; Huang H.; Zhang X. Synthesis and structure investigation of hexamethylene diisocyanate (HDI)-based polyisocyanates. Res. Chem. Intermed. 2017, 43 (5), 2799–2816. 10.1007/s11164-016-2795-1.

[ref53] Jiricny J.; Reese C. B. The Thermal Decomposition of Isocyanurates. Br. Polym. J. 1980, 12 (3), 81–84. 10.1002/pi.4980120302.

[ref54] Ketata N.; Sanglar C.; Waton H.; Alamercery S.; Delolme F.; Raffin G.; Grenier-Loustalot M. F. Thermal Degradation of Polyurethane Bicomponent Systems in Controlled Atmospheres. Polym. Polym. Compos. 2005, 13 (1), 1–26. 10.1177/096739110501300101.

[ref55] Jiao L.; Xiao H.; Wang Q.; Sun J. Thermal degradation characteristics of rigid polyurethane foam and the volatile products analysis with TG-FTIR-MS. Polym. Degrad. Stab. 2013, 98 (12), 2687–2696. 10.1016/j.polymdegradstab.2013.09.032.

[ref56] Wu G.; An J.; Tang X.-Z.; Xiang Y.; Yang J. A Versatile Approach towards Multifunctional Robust Microcapsules with Tunable, Restorable, and Solvent-Proof Superhydrophobicity for Self-Healing and Self-Cleaning Coatings. Adv. Funct. Mater. 2014, 24 (43), 6751–6761. 10.1002/adfm.201401473.

[ref57] Huang M.; Yang J. Facile microencapsulation of HDI for self-healing anticorrosion coatings. J. Mater. Chem. 2011, 21 (30), 11123–11130. 10.1039/c1jm10794a.

[ref58] Wu G.; An J.; Sun D.; Tang X.; Xiang Y.; Yang J. Robust microcapsules with polyurea/silica hybrid shell for one-part self-healing anticorrosion coatings. J. Mater. Chem. A 2014, 2 (30), 11614–11620. 10.1039/C4TA01312C.

[ref59] Sun D.; An J.; Wu G.; Yang J. Double-layered reactive microcapsules with excellent thermal and non-polar solvent resistance for self-healing coatings. J. Mater. Chem. A 2015, 3 (8), 4435–4444. 10.1039/C4TA05339G.

[ref60] Kothari J.; Iroh J. O. Self-Healing Poly(urea formaldehyde) Microcapsules: Synthesis and Characterization. Polymers 2023, 15 (7), 1668 10.3390/polym15071668. 37050281 PMC10096928

[ref61] Nguyen L.-T. T.; Hillewaere X. K. D.; Teixeira R. F. A.; van den Berg O.; Du Prez F. E. Efficient microencapsulation of a liquid isocyanate with in situ shell functionalization. Polym. Chem. 2015, 6 (7), 1159–1170. 10.1039/C4PY01448K.

[ref62] Aguiar A.; Loureiro M. V.; Pinho I.; Marques A. C. Efficient encapsulation of isocyanates in PCL/PLA biodegradable microcapsules for adhesives. J. Mater. Sci. 2023, 58 (5), 2249–2267. 10.1007/s10853-023-08160-9.

[ref63] Orgilés-Calpena E.; Arán-Aís F.; Torró-Palau A. M.; Sánchez M. A. M. Adhesives in the footwear industry: a critical review. Rev. Adhes. Adhes. 2019, 7 (1), 69–71. 10.7569/RAA.2019.097303.

[ref64] Bamber N.; Turner I.; Arulnathan V.; Li Y.; Zargar Ershadi S.; Smart A.; Pelletier N. Comparing sources and analysis of uncertainty in consequential and attributional life cycle assessment: review of current practice and recommendations. Int. J. Life Cycle Assess. 2020, 25 (1), 168–180. 10.1007/s11367-019-01663-1.

