# Supplementary material for: Microcapsules of Poly(butylene adipate-co-terephthalate) (PBAT) Loaded with Aliphatic Isocyanates for Adhesive Applications
Source: ACS Appl Polym Mater. 2024 May 2;6(10):5618–29. doi: 10.1021/acsapm.4c00033 (PMC11129179; doi:10.1021/acsapm.4c00033)
Supplement: Supplementary file 1 — ap4c00033_si_001.pdf [file ap4c00033_si_001.pdf]

Supporting Information

Microcapsules of poly(butylene adipate-co-terephthalate) (PBAT) loaded with aliphatic isocyanates for adhesive applications

António Aguiar [a]*, Lucas P. Marcelino[a], António Mariquito[a], Carla L. Simões[b], Ricardo Simoes[b,c], Isabel Pinho[d] and Ana C. Marques[a]*

[a]*CERENA, DEQ, Instituto Superior Técnico, Universidade de Lisboa, Avenida Rovisco Pais, 1049-001 Lisboa, Portugal*
[b]*Polytechnic Institute of Cavado and Ave (IPCA), 4750-810 Barcelos, Portugal*
[c]*Institute for Polymers and Composites (IPC), University of Minho, 4800-058 Guimarães, Portugal*
[d]*CIPADE,* Av. Primeiro de Maio 121, 3700-227 São João da Madeira, Portugal.

*Email: antonio.luis.aguiar@tecnico.ulisboa.pt; ana.marques@tecnico.ulisboa.pt

Contents

Figure S1. NMR spectra, in $CDCl_3$, and calibration curve for PHDI quantification S2

Table S1. Inventory results of CA adhesive system to 1 kg of adhesive (FU). S3

Table S2. Inventory results of MCA system to 1 kg of adhesive (FU). S4

Figure S2. Examples of MCs where the isocyanate was not well protected and PUa layer was formed.. .... S5

Figure S3. MCs-TriHDI's aggregates. S5

Figure S4. Histogram size distribution and cumulative distribution (Q1=D25, Q2=D50, Q3=D75) of the MCs. S6

Figure S5. SEM image of the MC-TriHDI showing big MCs acting as containers for smaller MCs. S7

Figure S6. ^1H NMR spectra enlargement of MCs-HDI, MCs-TriHDI and MCs-PHDI. S7

Figure S7. FTIR spectra of PBAT, HDI, TriHDI and PHDI. S8

Figure S8. DTG of PBAT, HDI, TriHDI and PHDI. S8

Figure S9. ^1H NMR spectra of MCs-HDI (13.8 mg) + 4-chloro-3-methylphenol (7.2 mg), in $CDCl_3$. S9

Figure S10. ^1H NMR spectra of MCs-TriHDI (16.8 mg) + 4-chloro-3-methylphenol (4.73 mg), in $CDCl_3$. S9

Figure S11. ^1H NMR spectra of MCs-PHDI (14.2 mg) + 4-chloro-3-methylphenol, in $CDCl_3$. S10

Figure S12. Thermograms and derivative curves of MCs-HDI, and thermograms of MCs-TriHDI and MCs-PHDI after production and 3 months later. S10

Figure S13. FTIR spectra of MCs-HDI after production and 3 months later. S11

Figure S14. Thermograms of MCs-HDI and MCs-PHDI in ethyl acetate, toluene and hexane over time. S11

Figure S15. SEM image of MCs-HDI after dispersion in hexane. S12

Table S3. Viscosity measurements of the adhesive formulations over 7 days. S12

Figure S16. Adhesive formulations and substrates after the adhesive formulation application. S12

Table S4. Peel test results. S13

Figure S17. Photographs of substrate failure after peel test. S13

Table S5. Environmental assessment results of the CA and MCA adhesive systems. S14

Figure S18. Relative contributions (in %) for GWP and EC of the CA adhesive production stage. S14

Figure S19. Relative contributions for GWP and EC of the MCA production stage. 15

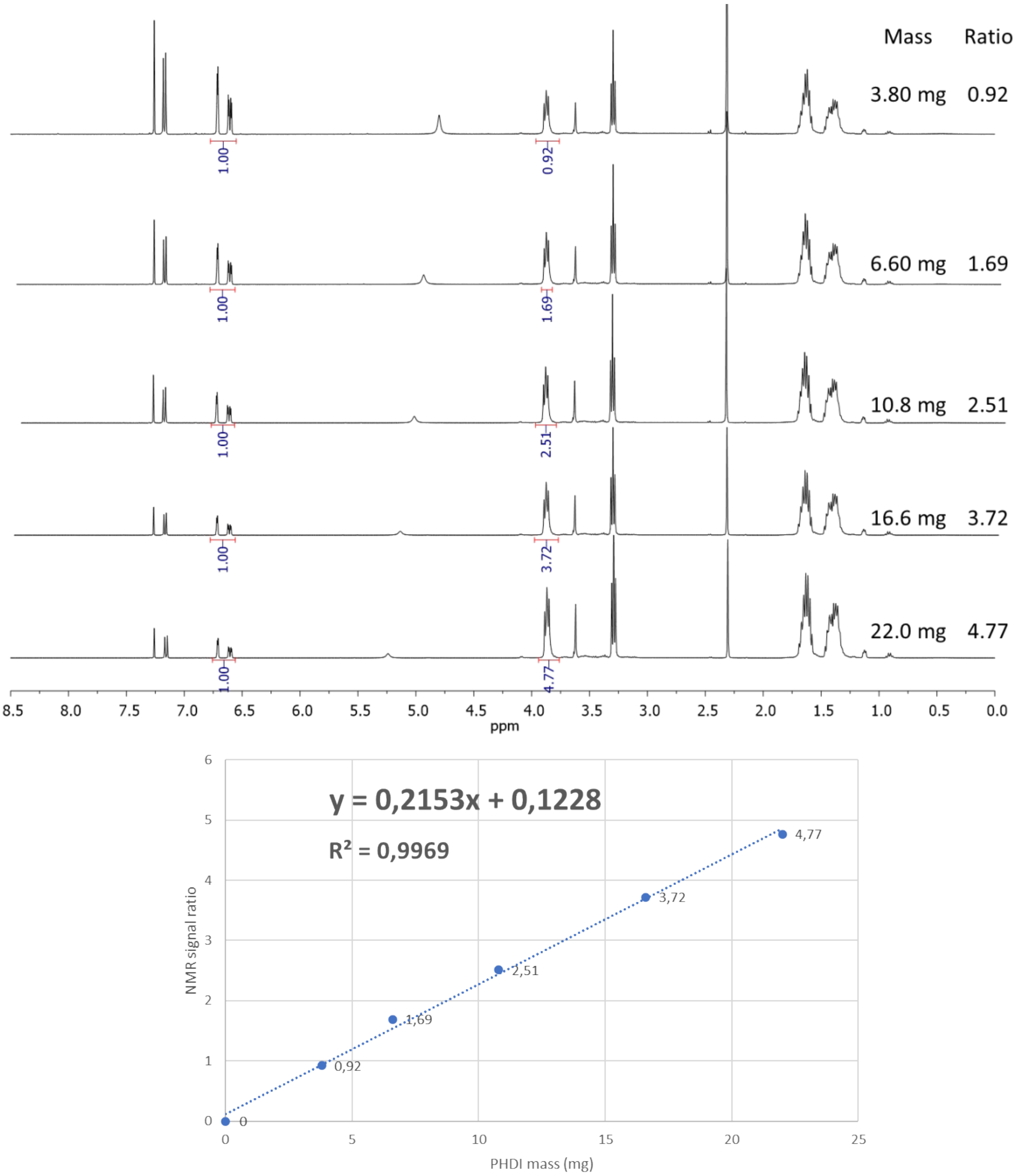

Figure S1. NMR spectra, in $CDCl_3$, and calibration curve for PHDI quantification

Table S1. Inventory results of CA adhesive system to 1 kg of adhesive (FU).

Flows	Amount/FU	Unit	Data source / Database
**Materials**			
Hydrophilic fumed silica	1.91E-02	kg	Primary data / Ecoinvent
Fumaric Acid	1.15E-03	kg	Primary data / Ecoinvent
Linear hydroxyl polyurethane	1.37E-01	kg	Primary data / Ecoinvent
High crystallization polyurethane	1.59E-02	kg	Primary data / Ecoinvent
Acetone	7.81E-01	kg	Primary data / Ecoinvent
Monopropylene glycol	9.55E-04	kg	Primary data / Ecoinvent
TDI isocyanate	3.60E-02	kg	Primary data / Ecoinvent
Ethyl acetate	9.01E-03	kg	Primary data / Ecoinvent
**CA Adhesive production**			
Transport of materials	879.253	Kg.km	Estimated / Ecoinvent
Energy (electricity)	0.085	kWh	Primary data / Ecoinvent
**CA Adhesive packaging**			
Tinplate	7.12E-02	kg	Primary data / ELCD
Polypropylene (PP)	4.95E-03	kg	Primary data / Ecoinvent
Production of tinplate packaging	7.12E-02	kg	Estimated / Ecoinvent
Production of PP packaging	4.95E-03	kg	Estimated / Ecoinvent
Packing transport	9.57E-01	Kg.km	Primary data / Ecoinvent
**CA Adhesive transport**			
CA adhesive transport	96.851	Kg.km	Estimated / Ecoinvent

Table S2. Inventory results of MCA system to 1 kg of adhesive (FU).

Flows	Amount/FU	Unit	Data source / Database
**Materials**			
Hydrophilic fumed silica	1.85E-02	kg	Primary data / Ecoinvent
Fumaric Acid	1.11E-03	kg	Primary data / Ecoinvent
Linear hydroxyl polyurethane	1.33E-01	kg	Primary data / Ecoinvent
High crystallization polyurethane	1.54E-02	kg	Primary data / Ecoinvent
Acetone	7.56E-01	kg	Primary data / Ecoinvent
Monopropylene glycol	9.25E-04	kg	Primary data / Ecoinvent
Polybutylene adipate terephthalate (PBAT)	3.25E-02	kg	Primary data / Ecoinvent
Dichloromethane (DCM)	8.79E-02	kg	Primary data / Ecoinvent
Isocyanate	6.88E-02	kg	Primary data / Industry data 2.0
Poly (vinyl alcohol) (PVA)	5.00E-03	kg	Primary data / Ecoinvent
Water	5.00E-01	kg	Primary data / Ecoinvent
Anti-foam	2.50E-03	kg	Primary data / Ecoinvent
Washing water	5.00E-01	kg	Primary data / Ecoinvent
**MCA production**			
Transport of materials	776.353	Kg.km	Estimated / Ecoinvent
Energy (electricity)	0.2	kWh	Primary data / Ecoinvent
**MCA packaging**			
Tinplate	6.89E-02	kg	Primary data / ELCD
Polyethylene terephthalate (PET)	9.60E-03	kg	Primary data / Ecoinvent
Production of tinplate packaging	6.89E-02	kg	Estimated / Ecoinvent
Production of PET packaging	9.60E-03	kg	Estimated / Ecoinvent
Packing transport	1.65	Kg.km	Primary data / Ecoinvent
**MCA transport**			
MCA transport	97.068	Kg.km	Estimated / Ecoinvent

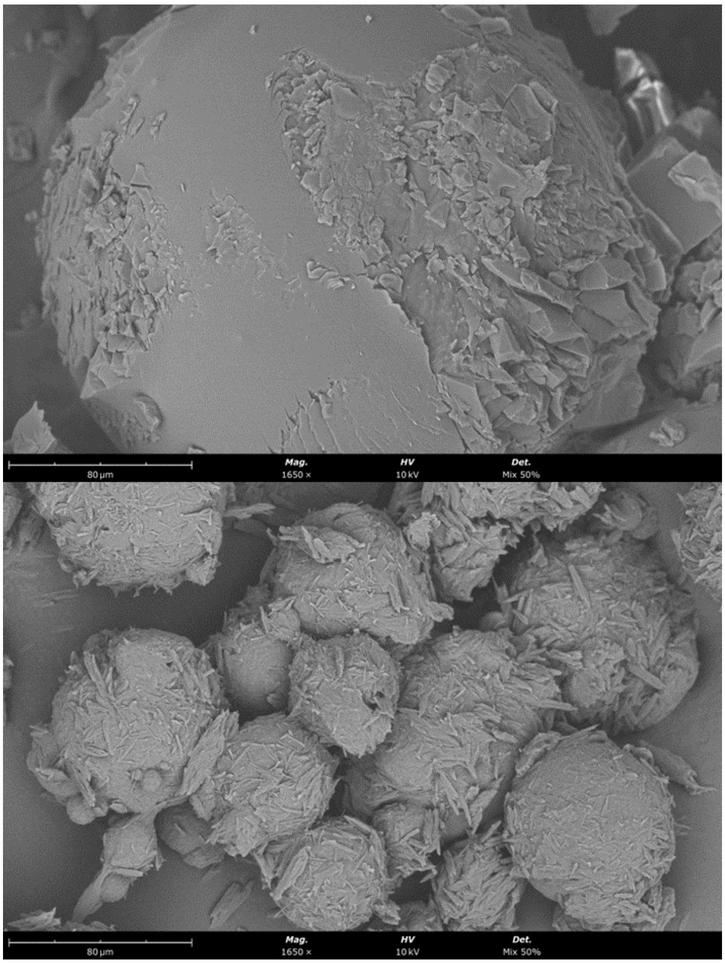

Figure S2. Examples of PBAT MCs where the isocyanate was not very well protected and PUa outer layer was formed. These MCs were not studied in the article and only serve as an example of flawed encapsulation processes.

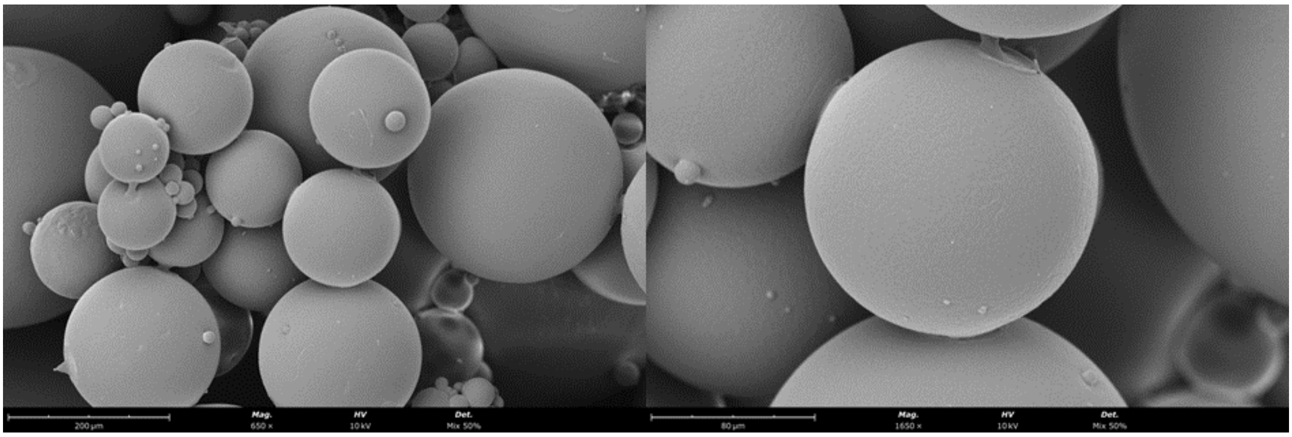

Figure S3. MCs-TriHDI’s aggregates.

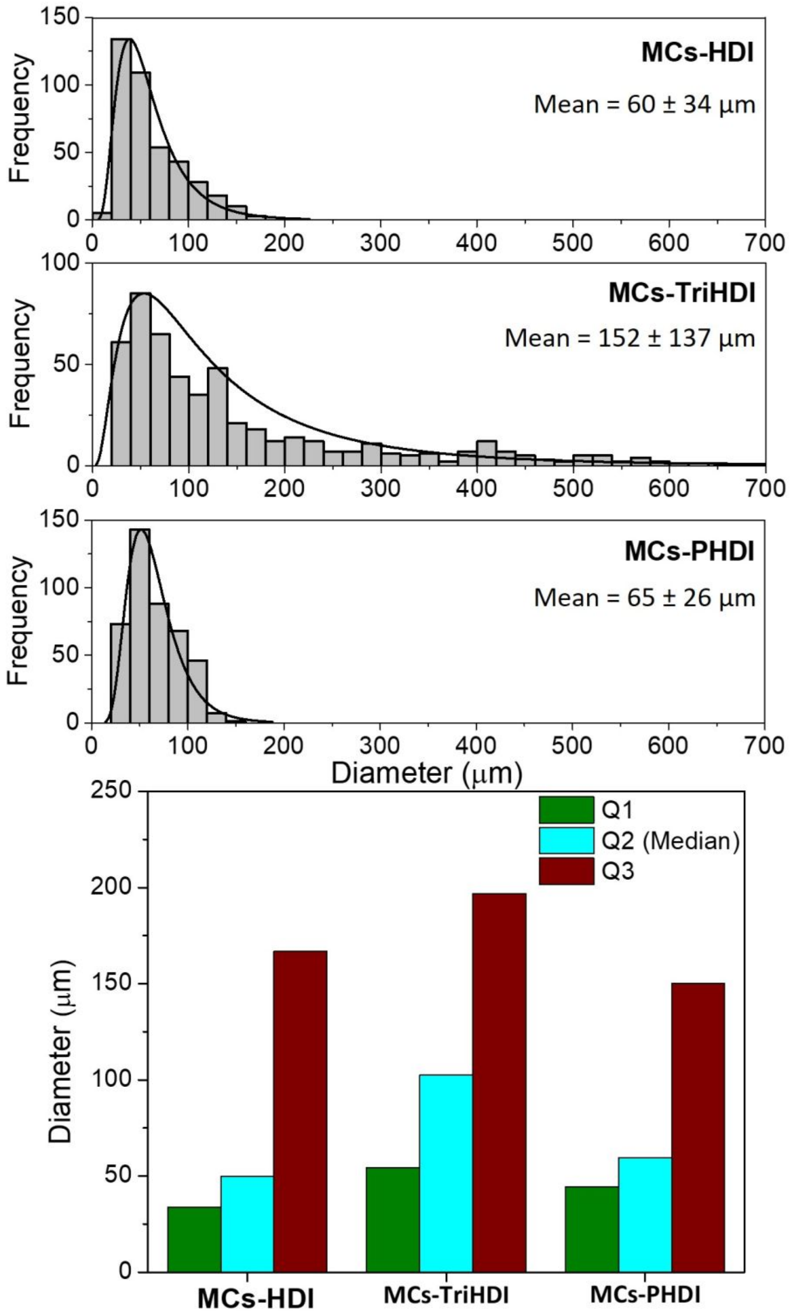

Figure S4. Histogram size distribution and cumulative distribution (Q1=D25, Q2=D50, Q3=D75) of the MCs.

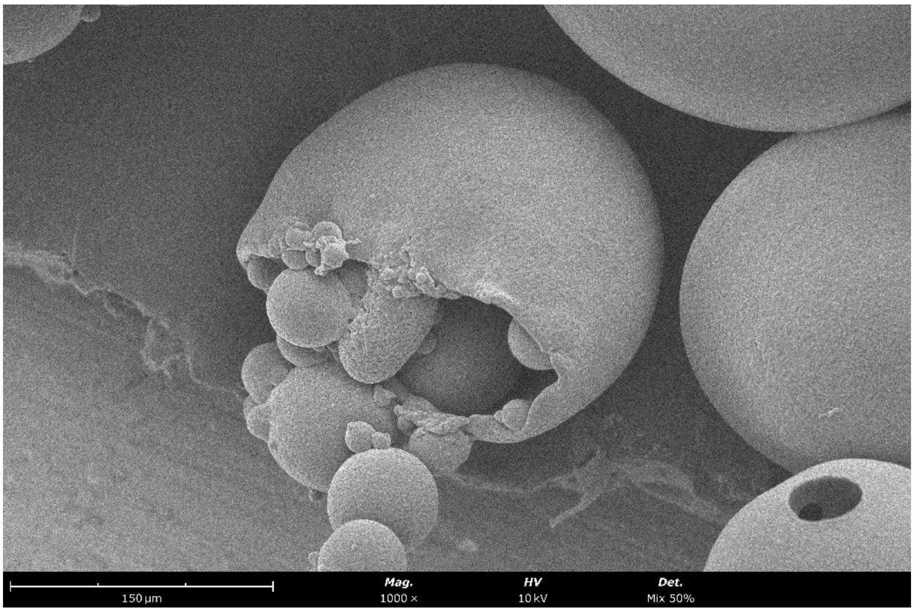

Figure S5. SEM image of the MC-TriHDI showing big MCs acting as containers for smaller MCs.

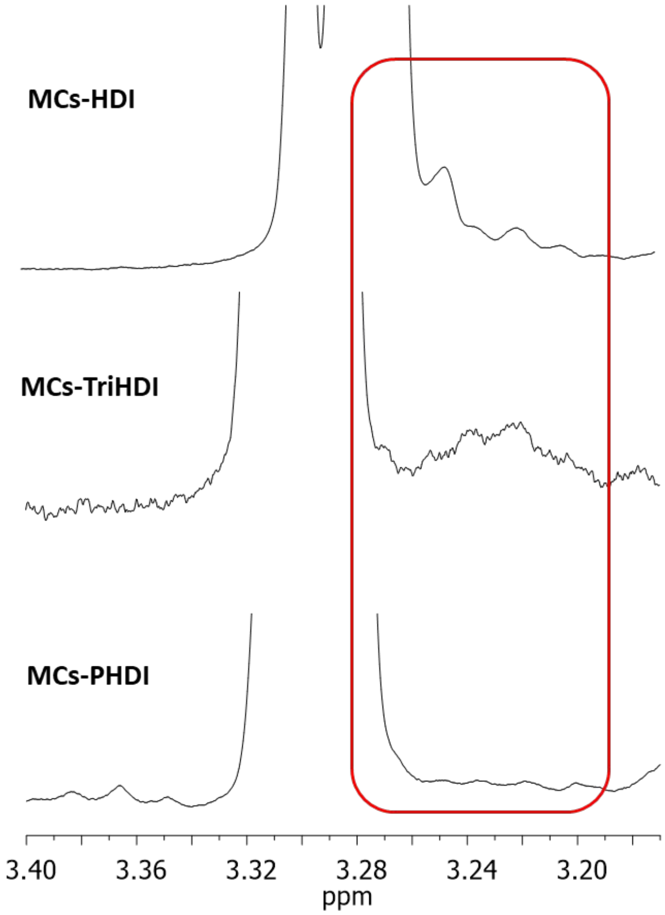

Figure S6. ^{1}H NMR spectra enlargement of MCs-HDI, MCs-TriHDI and MCs-PHDI to show a signal caused by polyurea formation.

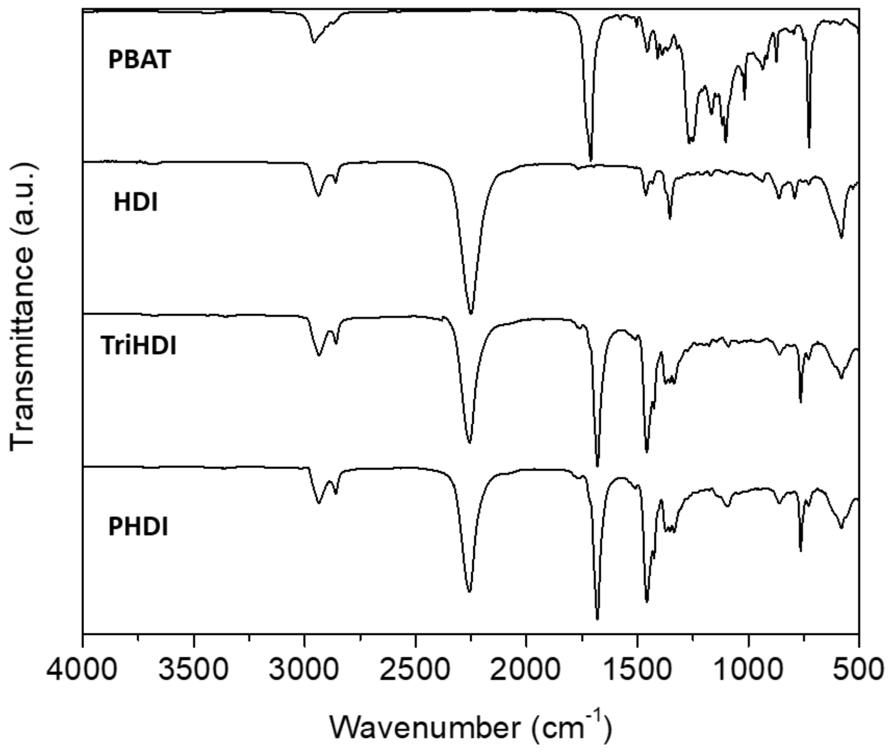

Figure S7. FTIR spectra of PBAT, HDI, TriHDI and PHDI.

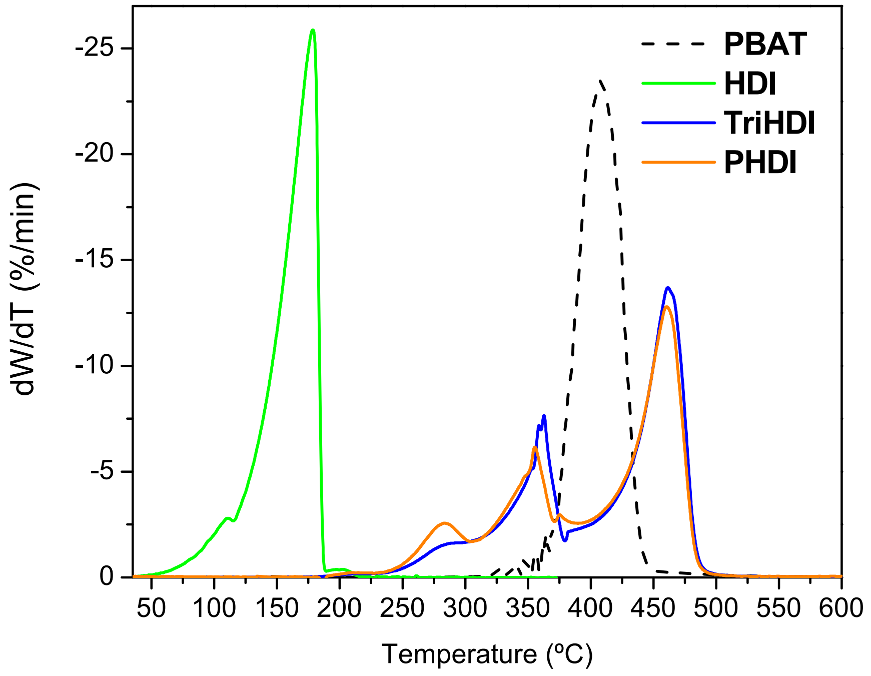

Figure S8. DTG of PBAT, HDI, TriHDI and PHDI.

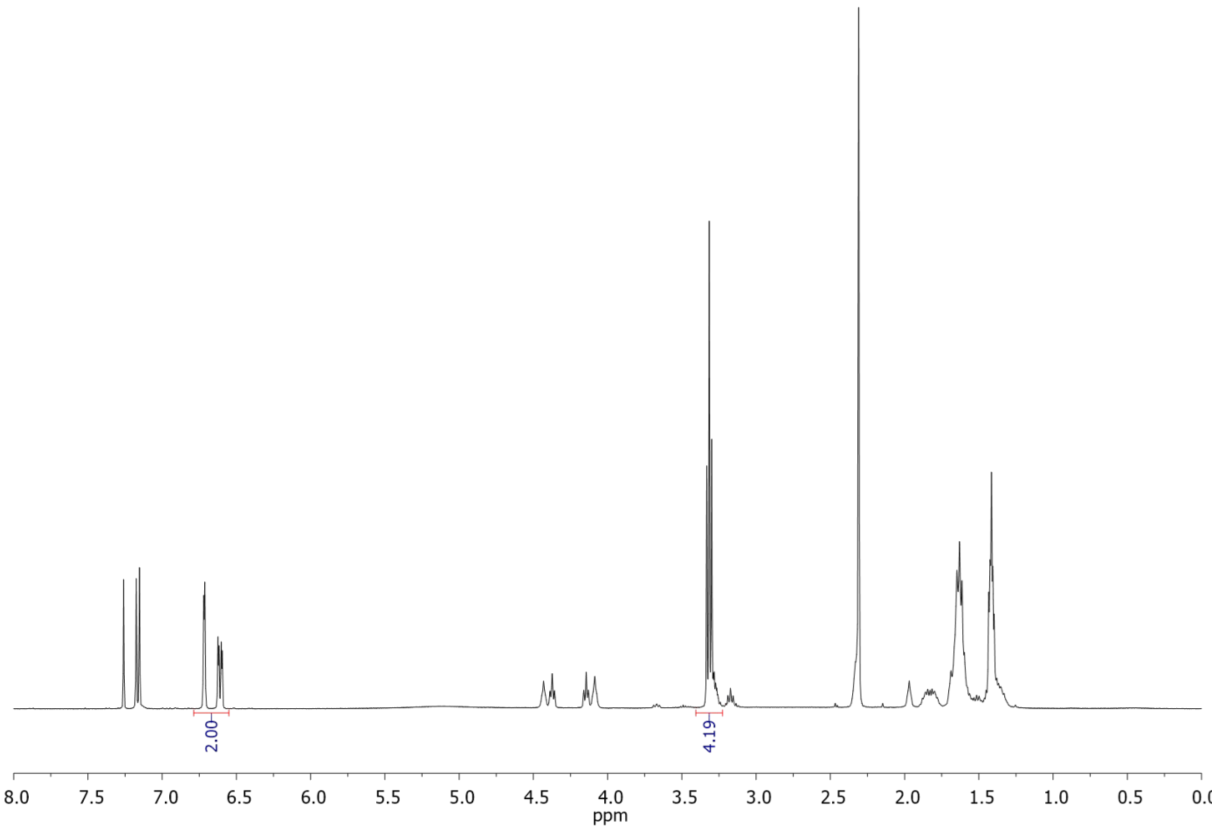

Figure S9. ^{1}H NMR spectra of MCs-HDI (13.8 mg) + 4-chloro-3-methylphenol (7.2 mg), in $CDCl_3$.

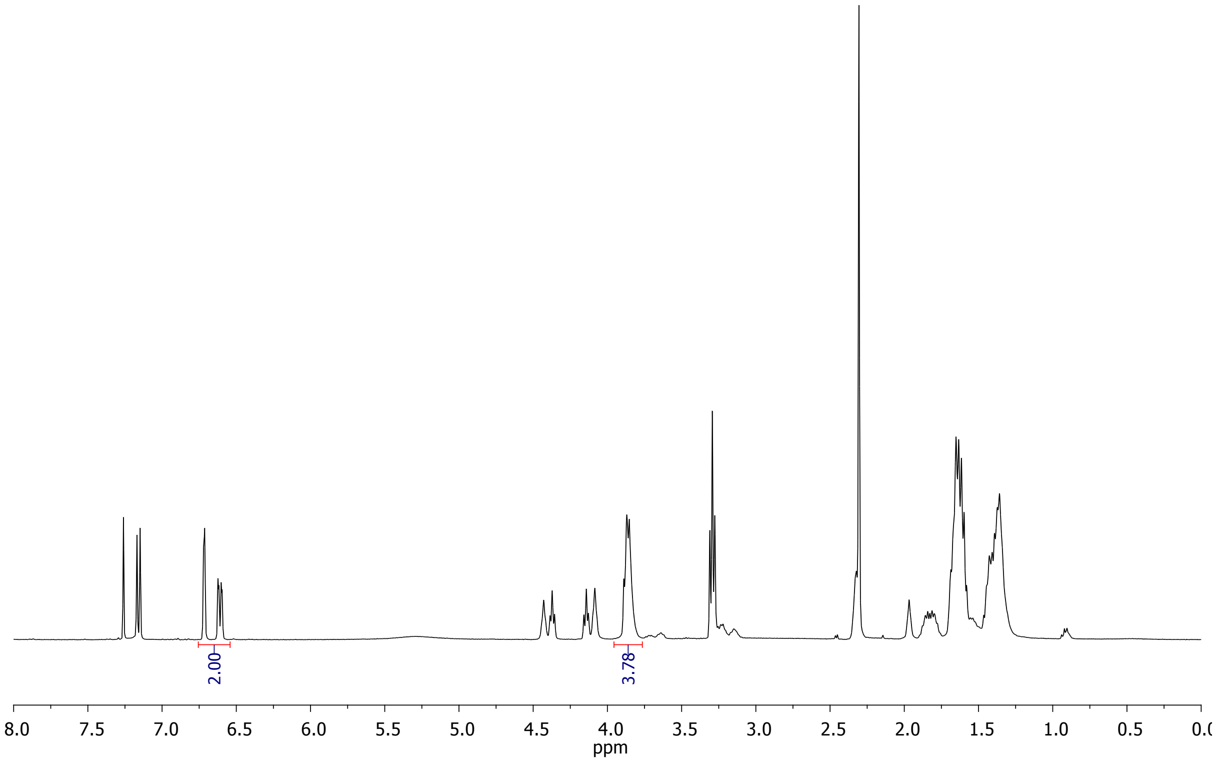

Figure S10. ^{1}H NMR spectra of MCs-TriHDI (16.8 mg) + 4-chloro-3-methylphenol (4.73 mg), in $CDCl_3$.

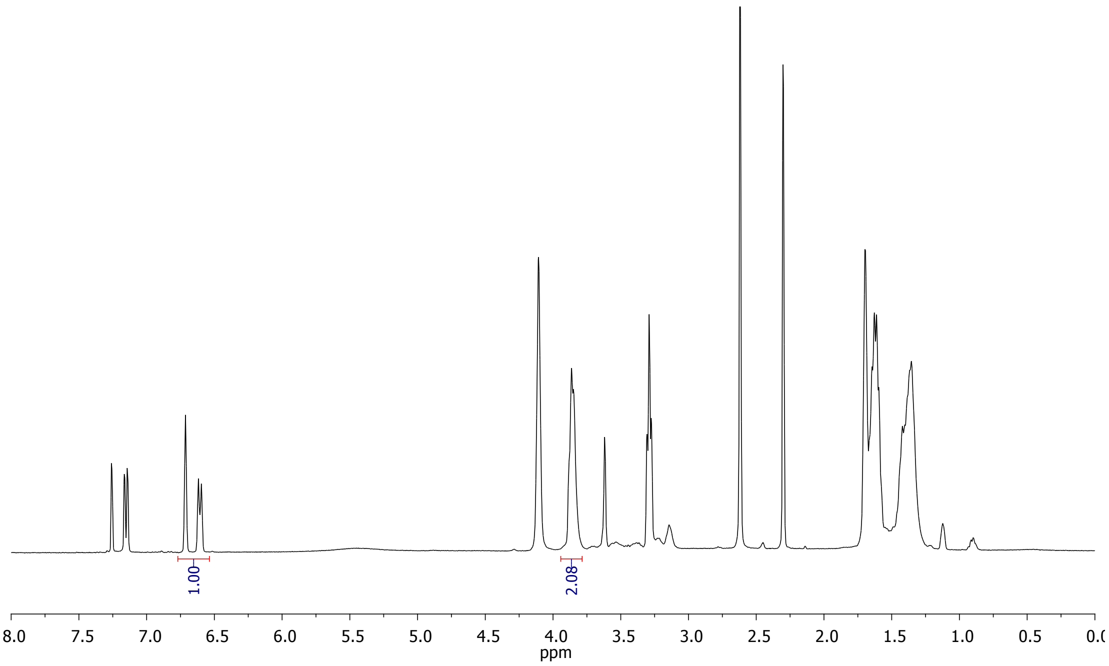

Figure S11. ^{1}H NMR spectra of MCs-PHDI (14.2 mg) + 4-chloro-3-methylphenol, in $CDCl_3$.

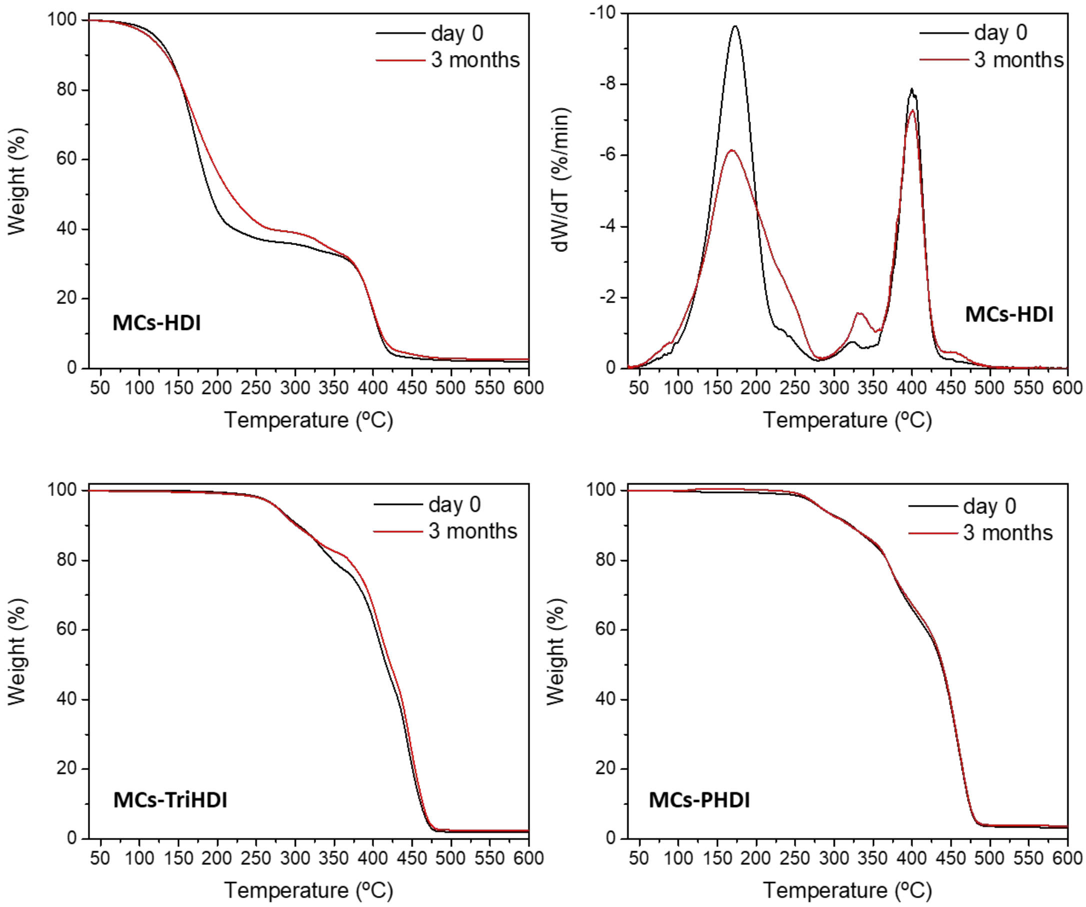

Figure S12. Thermograms and derivative curves of MCs-HDI, and thermograms of MCs-TriHDI and MCs-PHDI after production and 3 months later.

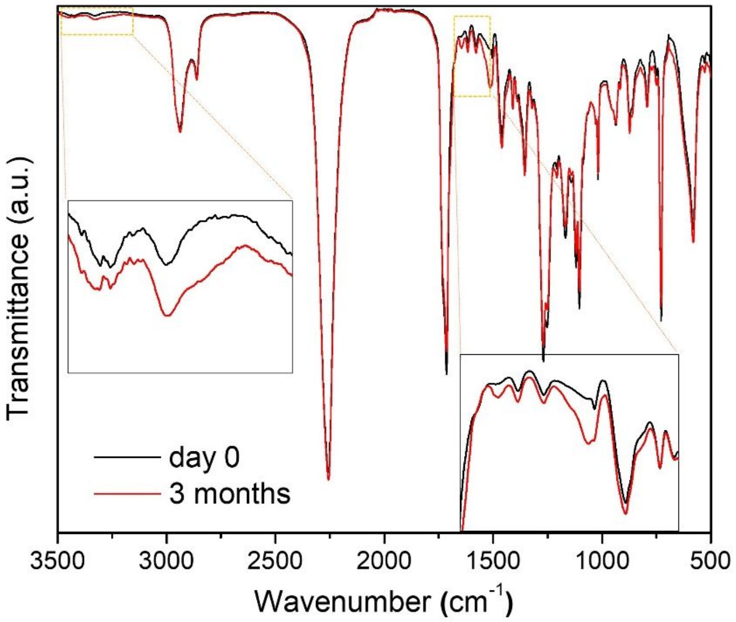

Figure S13. FTIR spectra of MCs-HDI after production and 3 months later.

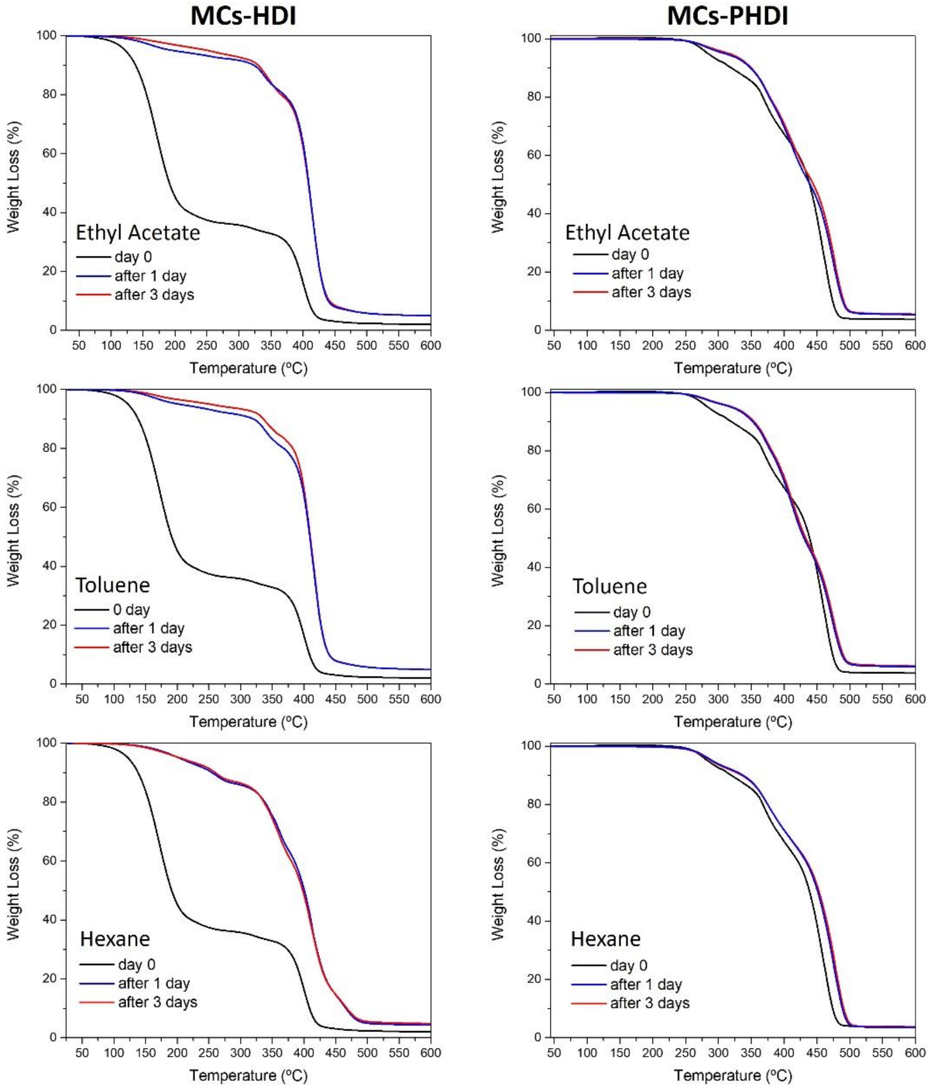

Figure S14. Thermograms of MCs-HDI and MCs-PHDI in ethyl acetate, toluene and hexane over time.

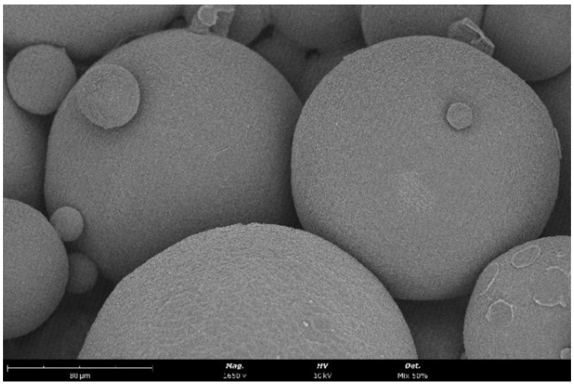

Figure S15. SEM image of MCs-HDI after dispersion in hexane.

Table S3. Viscosity measurements of the adhesive formulations over 7 days.

Sample	Day 0	Day 1	Day 2	Day 4	Day 7
**6275**	3.84 ± 0.26 Pa.s	5.45 ± 0.13 Pa.s	5.77 ± 0.27 Pa.s	5.78 ± 0.05 Pa.s	8.15 ± 0.41 Pa.s
**6275 + HDI**	3.84 ± 0.26 Pa.s	10.66 ± 0.69 Pa.s	11.7 ± 0.23 Pa.s	34.37 ± 4.62 Pa.s	47.35 ± 2.58 Pa.s
**6275 + TriHDI**	3.84 ± 0.26 Pa.s	11.61 ± 0.8 Pa.s	11.74 ± 0.23 Pa.s	25.98 ± 1.49 Pa.s	39.27 ± 2.48 Pa.s
**6275 + PHDI**	3.84 ± 0.26 Pa.s	10.92 ± 0.8 Pa.s	11.53 ± 0.65 Pa.s	21.17 ± 3.16 Pa.s	42.15 ± 1.93 Pa.s
**6275 + MCs-HDI**	3.84 ± 0.26 Pa.s	10.95 ± 0.47 Pa.s	11.95 ± 0.49 Pa.s	12.79 ± 0.92 Pa.s	27.29 ± 2.31 Pa.s
**6275 + MCs-TriHDI**	3.84 ± 0.26 Pa.s	13.06 ± 1.16 Pa.s	15.01 ± 0.17 Pa.s	22.11 ± 1.25 Pa.s	42.34 ± 3.12 Pa.s
**6275 + MCs-PHDI**	3.84 ± 0.26 Pa.s	11.52 ± 0.36 Pa.s	13.06 ± 0.85 Pa.s	18.67 ± 1.3 Pa.s	35.32 ± 4.69 Pa.s

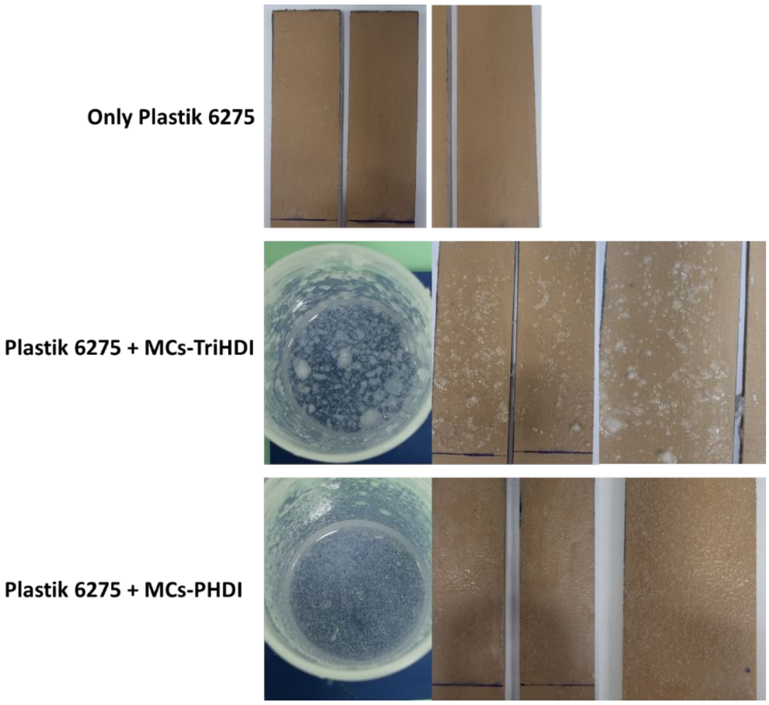

Figure S16. Adhesive formulations and substrates after the adhesive formulation application.

Table S4. Peel test results.

Sample	Average force/width (N/mm)	Maximum strength (N)	Average Strength (N)	Average force/width (N/mm)
**6275**	5.3	162.14	118.76	4.84 ± 0.43
	4.94	237.97	185.66	
	4.27	269.36	238.05	
**6275 + HDI**	3.92	149.06	117.58	4.01 ± 0.09
	4.09	154.29	122.81	
**6275 + TriHDI**	7.04	273.28	211.18	6.16 ± 0.64
	5.55	189.60	166.62	
	5.89	215.75	176.84	
**6275 + PHDI**	4.66	175.21	139.84	4.90 ± 0.37
	4.61	103.30	78.27	
	5.42	176.52	132.68	
**6275 + MCs-HDI**	4.64	193.52	139.15	5.34 ± 1.89
	3.45	141.22	103.48	
	7.93	308.58	237.84	
**6275 + MCs-TriHDI**	4.30	146.45	129.03	4.19 ± 1.02
	5.38	176.52	161.41	
	2.90	188.29	86.98	
**6275 + MCs-PHDI**	5.43	193.52	162.99	4.82 ± 0.45
	4.36	201.36	130.95	
	4.66	209.21	139.76	

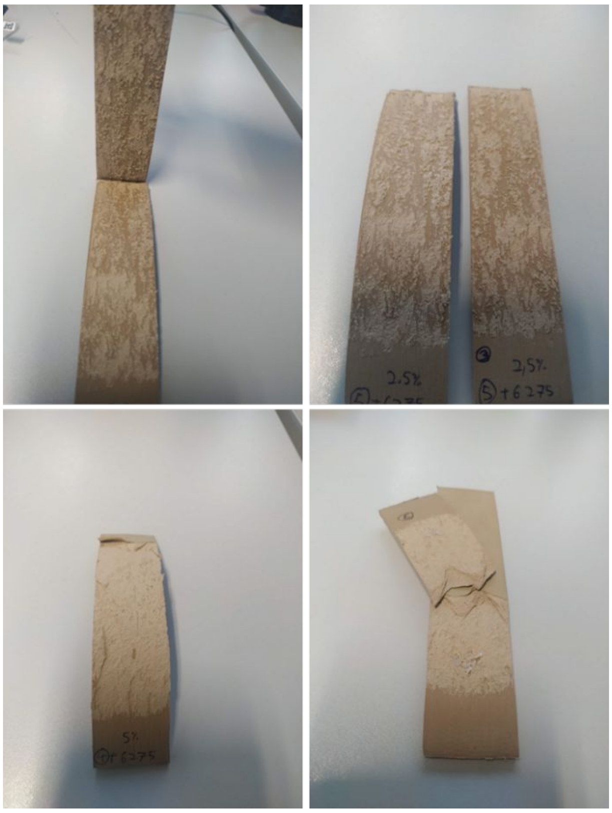

Figure S17. Photographs of substrate failure after peel test.

Table S5. Environmental assessment results of the CA and MCA adhesive systems.

System	Processes	Global Warming Potential ($kg\ CO_2\ eq$)	Energy consumption (non-renewable fossil) (MJ primary)
CA	Production	2.397	72.315
	Packaging	0.183	2.462
	Transport	0.009	0.149
	Total	2.588	74.926
MCA	Production	2.881	80.900
	Packaging	0.201	2.872
	Transport	0.009	0.149
	Total	3.090	83.920

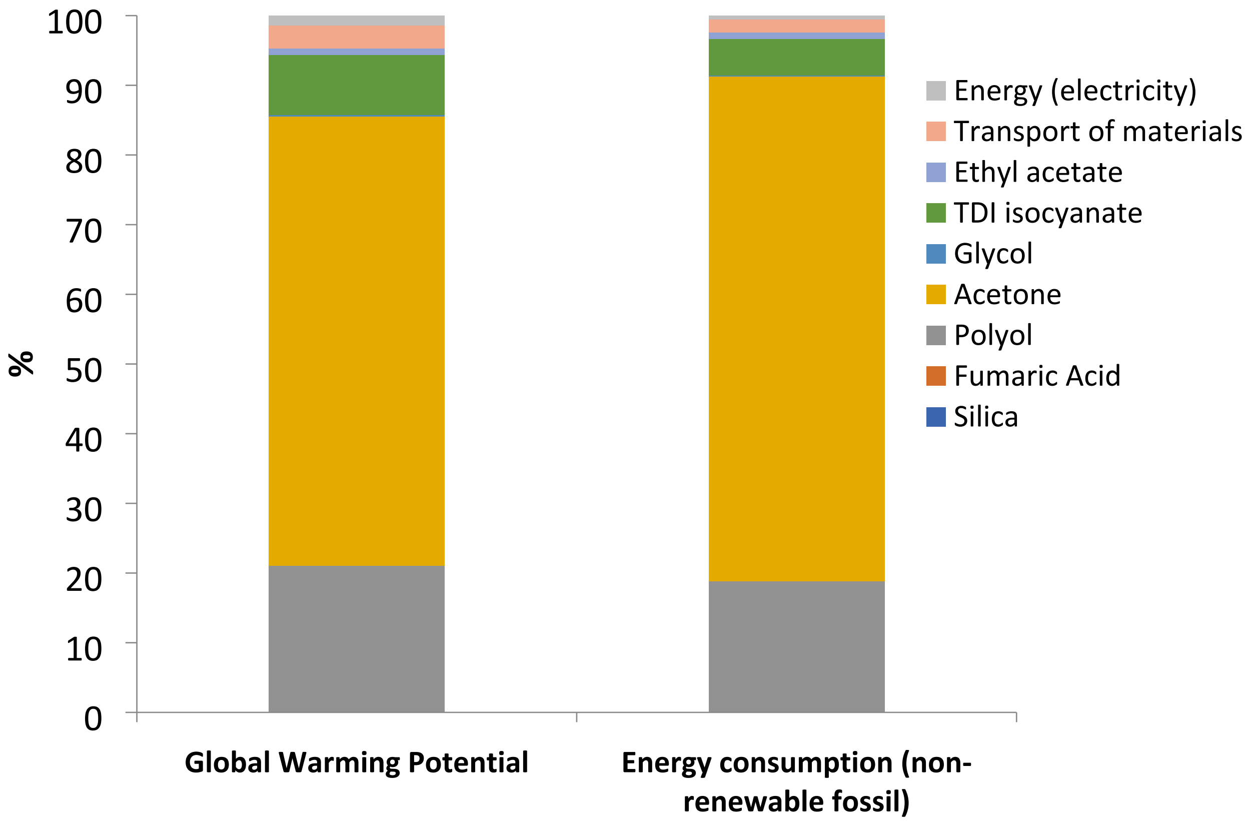

Figure S18. Relative contributions (in %) for GWP and EC of the CA adhesive production stage.

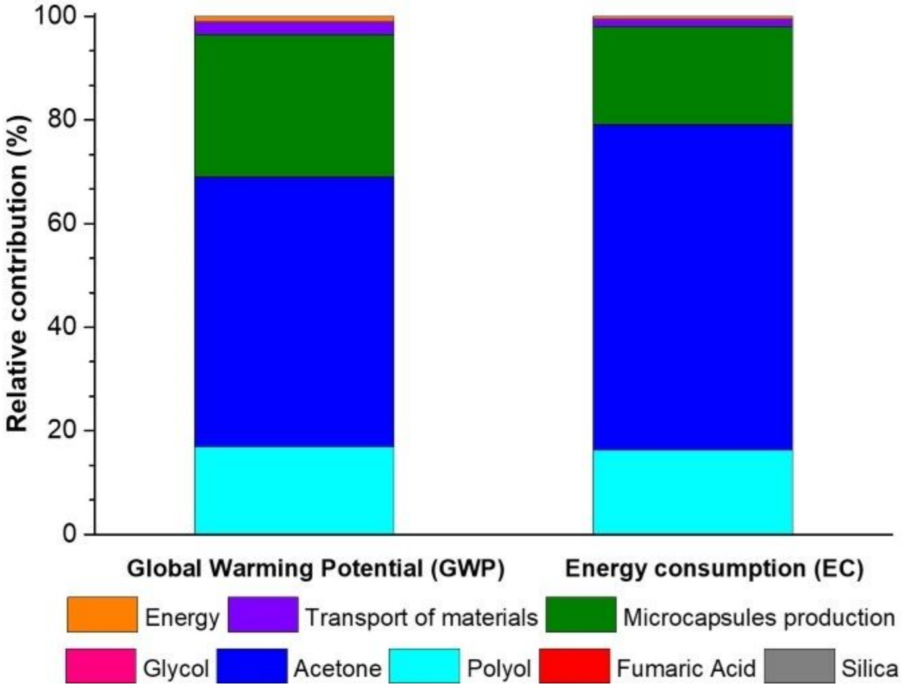

Figure S19. Relative contributions for GWP and EC of the MCA production stage.
